# The PIWI-interacting protein Gtsf1 controls the selective degradation of small RNAs in *Paramecium*

**DOI:** 10.1093/nar/gkae1055

**Published:** 2024-11-22

**Authors:** Olivia Charmant, Julita Gruchota, Olivier Arnaiz, Katarzyna P Nowak, Nicolas Moisan, Coralie Zangarelli, Mireille Bétermier, Anna Anielska-Mazur, Véronique Legros, Guillaume Chevreux, Jacek K Nowak, Sandra Duharcourt

**Affiliations:** Université Paris Cité, CNRS, Institut Jacques Monod, 15 rue Hélène Brion, F-75013 Paris, France; Institute of Biochemistry and Biophysics Polish Academy of Sciences, Pawinskiego 5a, 02-106 Warsaw, Poland; Université Paris-Saclay, CEA, CNRS, Institute for Integrative Biology of the Cell (I2BC), 1 Avenue de la Terrasse, 91198 Gif-sur-Yvette, France; Institute of Biochemistry and Biophysics Polish Academy of Sciences, Pawinskiego 5a, 02-106 Warsaw, Poland; Université Paris Cité, CNRS, Institut Jacques Monod, 15 rue Hélène Brion, F-75013 Paris, France; Université Paris-Saclay, CEA, CNRS, Institute for Integrative Biology of the Cell (I2BC), 1 Avenue de la Terrasse, 91198 Gif-sur-Yvette, France; Université Paris-Saclay, CEA, CNRS, Institute for Integrative Biology of the Cell (I2BC), 1 Avenue de la Terrasse, 91198 Gif-sur-Yvette, France; Institute of Biochemistry and Biophysics Polish Academy of Sciences, Pawinskiego 5a, 02-106 Warsaw, Poland; Université Paris Cité, CNRS, Institut Jacques Monod, 15 rue Hélène Brion, F-75013 Paris, France; Université Paris Cité, CNRS, Institut Jacques Monod, 15 rue Hélène Brion, F-75013 Paris, France; Institute of Biochemistry and Biophysics Polish Academy of Sciences, Pawinskiego 5a, 02-106 Warsaw, Poland; Université Paris Cité, CNRS, Institut Jacques Monod, 15 rue Hélène Brion, F-75013 Paris, France

## Abstract

Ciliates undergo developmentally programmed genome elimination, in which small RNAs direct the removal of transposable elements (TEs) during the development of the somatic nucleus. Twenty-five nucleotide scanRNAs (scnRNAs) are produced from the entire germline genome and transported to the maternal somatic nucleus, where selection of scnRNAs corresponding to germline-specific sequences is thought to take place. Selected scnRNAs then guide the elimination of TEs in the developing somatic nucleus. How germline-specific scnRNAs are selected remains to be determined. Here, we provide important mechanistic insights into the scnRNA selection pathway by identifying a *Paramecium* homolog of Gtsf1 as essential for the selective degradation of scnRNAs corresponding to retained somatic sequences. Consistently, we also show that Gtsf1 is localized in the maternal somatic nucleus where it associates with the scnRNA-binding protein Ptiwi09. Furthermore, we demonstrate that the scnRNA selection process is critical for genome elimination. We propose that Gtsf1 is required for the coordinated degradation of Ptiwi09-scnRNA complexes that pair with target RNA via the ubiquitin pathway, similarly to the mechanism suggested for microRNA target-directed degradation in metazoans.

## Graphical Abstract

**Graphical Abstract:**
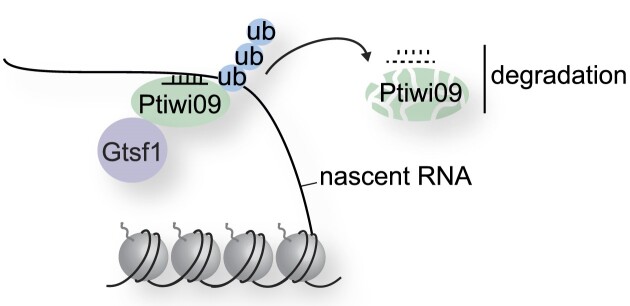

## Introduction

One major host defense mechanism to silence transposable elements (TE) is the small RNA silencing pathway. Small RNAs of 20–30 nt in length are loaded onto PIWI proteins to form small RNA–PIWI silencing complexes, which pair with nascent transcript by sequence complementarity, recruit histone methyltransferases to chromatin and repress the transcriptional activity of TEs and other repeats (1). Small RNA sequences are highly diverse and yet highly specific to TEs. The mechanisms underlying the distinction between TEs and the rest of the genome during the establishment of small RNA populations remain to be fully understood.

In animal gonadal cells, such as the *Drosophila* ovaries, Piwi-interacting RNAs (piRNAs) are mostly derived from heterochromatic TE-rich loci known as the piRNA clusters, and cluster transcripts must be specifically selected for piRNA biogenesis (2). Thus, the vast majority of these small RNAs originate from distinct genomic hotspots. The logic is radically different in the ciliate *Paramecium*, in which the entire germline genome initially produces 25-nt scnRNAs from both TE and non-TE sequences (3–6). In subsequent steps, the subpopulation of scnRNAs that correspond to TEs is selected to trigger the physical removal of TEs from the genome, a definitive form of TE silencing (3,7–9). The mechanism that enables the specific selection of scnRNAs corresponding to TEs has yet to be fully elucidated.

In *Paramecium*, the germline and somatic functions are supported by two types of nuclei that coexist in the same cytoplasm. The diploid germline micronucleus (MIC) transmits the genetic information from one generation to the next, while the polyploid somatic macronucleus (MAC) ensures gene expression, but is destroyed at each sexual cycle. During the self-fertilization process of autogamy, the MIC undergoes meiosis and karyogamy to produce the zygotic nucleus. New MICs and new MACs develop from mitotic products of the zygotic nucleus. During development of the new MAC, massive and reproducible elimination of nearly 30% of the genome (∼30 Mb out of 108 Mb) of specific germline sequences occurs (10). Eliminated germline sequences include 45 000 Internal Eliminated Sequences (IESs) that are remnants of TEs scattered throughout the genome (11,12). Other eliminated sequences (OES) (13) correspond to large regions comprising repeats such as TEs and satellites (14). Understanding how such diverse sequences are defined and eliminated despite the lack of conserved sequence motifs remains challenging.

Previous studies have shown that the specific recognition of TEs destined for elimination in fact involves scnRNAs which direct histone mark deposition and subsequent DNA excision in the developing MAC genome (15,16). In a mechanism very similar to transcriptional TE silencing described in fungi and metazoans (17,18), scnRNAs target the Piwi proteins to complementary nascent transcripts (19) to guide histone modifications on TE loci in the new MAC (20), thereby providing the required sequence specificity. A physical interaction between the scnRNA-binding protein Ptiwi09 and the Polycomb Repressive Complex 2 (PRC2-Ezl1) was recently shown to mediate the establishment of histone H3K9me3 and H3K27me3 at scnRNA-targeted regions (16,21). Deposition of these repressive chromatin marks in the new MAC is essential for the elimination of TEs and of 70% of IESs (16,20,22).

Twenty-five nucleotide long scnRNAs are produced during meiosis from the whole germline MIC genome, which is entirely transcribed by RNA polymerase II with the participation of a specialized Spt5/Spt4 elongation complex (23–26). scnRNA biogenesis relies on a developmental-specific RNA interference (RNAi) pathway that involves the Dicer-like proteins Dcl2 and Dcl3 (3–6,26). These scnRNAs then bind to Ptiwi01/09 proteins and are transported to the maternal MAC (8), where selection of scnRNAs is believed to occur. Working models posit that scnRNAs are sorted out by pairing interactions with nascent non-coding RNAs produced by the MAC genome (9,27,28). Non-coding RNAs are thought to trigger the degradation of their cognate small RNA (MAC-scnRNAs), while allowing scnRNAs corresponding to MIC-specific sequences (thereafter called MIC-scnRNAs), which by definition cannot pair with MAC transcripts, to be retained. This selective degradation of MAC-scnRNAs would thus result in the specific selection of the subpopulation corresponding to MIC-scnRNAs. The requirement of complementary maternal MAC transcripts for scnRNA selection has been directly demonstrated (9). On the other hand, how MAC-scnRNAs are degraded and how scnRNAs corresponding to TEs are selected is currently unknown.

Approximately 60% of the scnRNA population (representing the 72 Mb corresponding to the MAC genome) is degraded, making *Paramecium* an exquisite model to decipher the underlying molecular mechanisms of this selective degradation. To uncover candidate proteins involved in the process, we sought to identify the protein partners of the scnRNA-binding protein Ptiwi09 during the developmental stage at which scnRNA selection is thought to occur. We discover that *Paramecium* Gtsf1 is a nuclear Ptiwi09-interacting protein in the maternal MAC. The conserved Asterix/Gametocyte-specific factor 1 (GTSF1) proteins are essential piRNA factors that contribute to the repression of transposons in mice, *Drosophila* and zebrafish (29). We show that *Paramecium* Gtsf1 is involved in scnRNA-guided TE repression by controlling the degradation of MAC-scnRNAs and of the Ptiwi09 protein. We further demonstrate that defective scnRNA degradation leads to histone modification and scnRNA-guided DNA elimination defects. We propose that Gtsf1 is required for the coordinated degradation of the Ptiwi09 proteins and the bound scnRNAs when engaged in paring interactions with complementary nascent maternal MAC RNA, similarly to the proposed mechanism for microRNA target-directed degradation.

## Materials and methods

### Paramecium strains, cultivation and autogamy

All experiments were carried out with the entirely homozygous strain 51 of *Paramecium tetraurelia*. Cells were grown in wheat grass powder (Pines International) infusion medium bacterized the day before use with *Klebsiella pneumoniae*, unless otherwise stated, and supplemented with 0.8 mg/ml β-sitosterol (Merck). Cultivation and autogamy were carried out at 27°C as described (30,31).

### Gene silencing experiments

Plasmids used for T7Pol-driven double-stranded RNA (dsRNA) production in silencing experiments were obtained by cloning PCR products from each gene using plasmid L4440 and *Escherichia coli* strain HT115 DE3, as previously described (Galvani and Sperling, 2002). Sequences used for silencing of *ICL7a*, *GTSF1*, *EZL1*, *PTIWI01*, *PTIWI09*, *PGM* and *EMA1* were segments 1–580 of PTET.51.1.G0700039 (*ICL7a*); 249–479 (*GTSF1*#1, pOC19) or 32–441 (*GTSF1*#2) of PTET.51.1.G0490019 (*GTSF1*); 989–1501 of PTET.51.1.G1740049 (*EZL1*) (22); 41–441 of PTET.51.1.G0710112 (*PTIWI01*) (7); 50–439 of PTET.51.1.G0660118 (*PTIWI09*) (7); 873–1440 of PTET.51.1.G0490162 (*PGM*) (32); 163–1176 of PTET.51.1.G0010313 (*EMA1a*/*PTMB.220*) (33). Because *EMA1a* and *EMA1b* (16) display 88% identity in the segment used for silencing (193 nt with 100% identity), the construct is likely to silence both *EMA1a* and *EMA1b* genes. Preparation of silencing medium and RNAi during autogamy were performed as described in (32). Lethality of post-autogamous cells after RNAi was assessed by transferring 30–60 individual post-autogamous cells to standard growth medium. Cells with a functional new MAC were identified as normally growing survivors unable to undergo a novel round of autogamy if starved after ∼8 divisions. Cells usually divided two to four times before dying upon *GTSF1* knockdown (KD) as for *PTIWI01/09* KD, and unlike *EZL1, EMA1a* or *PGM* KD cells, which usually did not divide or only did so once before dying. See Supplementary Table S1 for details on RNAi-mediated KD experiments.

### Cytological stages monitoring and description

Progression of autogamy was followed by cytology with DAPI or Hoechst staining in the time course experiments. The progression through autogamy is not synchronous in the cell population (34). The time points refer to hours after T = 0 h (the onset of autogamy) that is defined as 50% of cells are autogamous (approximately 25% have a fragmented maternal MAC), as evaluated by cytological observation. See Supplementary Figure S1 for details on progression of autogamy.

### Transformation with tagged transgenes

For the construction of in-frame *3xFLAG-HA-GTSF1* (pOC17), *3xFLAG-PTIWI09* [pJG091 (26)] fusion plasmids, 3xFLAG-HA or 3xFLAG tags that were codon-optimized for the *P. tetraurelia* genetic code were added to the 5′ of the gene. As a result, the tag is fused to the N-terminus of *GTSF1* or of *PTIWI09*. The fusion proteins are expressed under the control of their endogenous regulatory regions (promoter and 3′UTR). *GTSF1* contains 74-bp upstream and 134-bp downstream of its open reading frame, and *PTIWI09* 257-bp upstream and 204-bp downstream. The *3xFLAG-HA-GTSF1* fusion transgene is RNAi-resistant. The 247–480 DNA fragments of *GTSF1* coding sequence was replaced with synthetic DNA sequences (Eurofins Genomics) designed to maximize nucleotide sequence divergence with the endogenous genomic loci without modifying the amino acid sequences of the encoded proteins. *3xFLAG-HA* (pIC12; 3xFLAG-HA tag only under *EZL1* regulatory sequences) (16), *3xFLAG-HA-EZL1* (pCM10) (20) and *3xFLAG-HA-GTSF1* (pOC17) were linearized by XmnI, *3xFLAG-HA-PTIWI09* (pAH30) (16) by SpeI, and *3xFLAG-PTIWI09* (pJG091) by AhdI for microinjection into the MAC of vegetative cells. No lethality was observed in the post-autogamous progeny of injected cells, indicating that none of the fusion constructs interfered with the normal progression of autogamy.

### Immunoprecipitation

**Figure 1.**
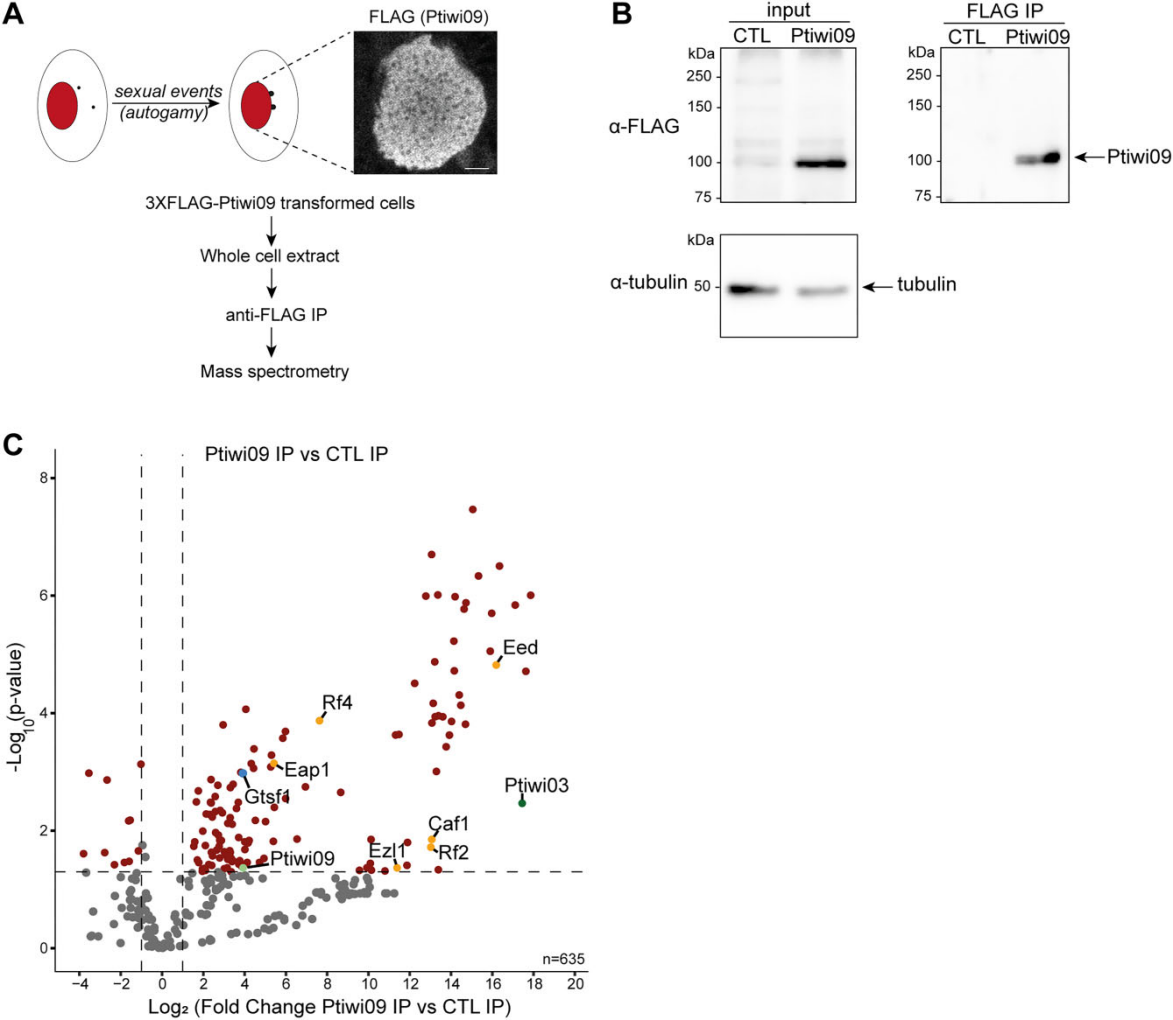
Identification of Ptiwi09-interacting partners in the maternal macronucleus. (**A**) Schematic representation of Ptiwi09 IP experiments. Anti-FLAG immunostaining of cells transformed with a *3XFLAG-PTIWI09* transgene when whole cell extracts for IP were performed (T = 0 h, at the onset of autogamy). Scale bar, 5 μm. (**B**) Western blot analysis of whole cell extracts from cells expressing *3XFLAG-PTIWI09* (Ptiwi09) and non-injected cells (control; CTL) before (input) or after affinity purification (immunoprecipitation; IP). FLAG antibodies were used for 3XFLAG-Ptiwi09 detection and tubulin antibodies for normalization. (**C**) Volcano plot of the quantitative label-free mass spectrometry (MS) analysis of 3XFLAG-Ptiwi09 affinity purification. Significantly enriched proteins in Ptiwi09 IP (four replicates) over control IP (two replicates) (statistical *t*-test, the fold change is greater than 2, the total number of peptides is greater than 2 and the *P*-value is less than 0.05) are in the upper right corner. The bait Ptiwi09, Gtsf1, PRC2-Ezl1 complex and Ptiwi09-interacting proteins are highlighted.

Ptiwi09 immunoprecipitation (IP) (Figure 1 and Supplementary Table S2) was performed as follows: *Paramecium* cells transformed with the 3x*FLAG-PTIWI09* transgene as well as non-transformed cells were grown until ∼1.5 h before T = 0 h. Two hundred milliliter of cells (4000 cells/ml) were frozen in liquid nitrogen. The cell pellet (300 μl) was resuspended in 3 ml lysis buffer [50mM Tris (pH = 7.4), 300 mM NaCl, 2 mM MgCl_2_, 10% glycerol, 2 mM ethylenediaminetetraacetic acid (EDTA), 0.3% Triton X-100, 2 mM phenylmethylsulfonyl fluoride (PMSF), 1× Pierce Protease Inhibitor Tablets, EDTA-Free], kept on ice for 20 min then lysed with a Potter-Elvehjem homogenizer. To whole cell lysate is incubated with (100 U) TURBO™ DNase (Ambion) for 30 min at 4°C then centrifuged for 30 min at 20 817 g at 4°C. The supernatant was incubated with 40 μl anti-FLAG M2 agarose gel (A2220, Sigma) for 4 h at 4°C. Beads were washed five times with lysis buffer and five times with wash buffer [10 mM Tris (pH = 7.4), 150 mM NaCl].

**Figure 2.**
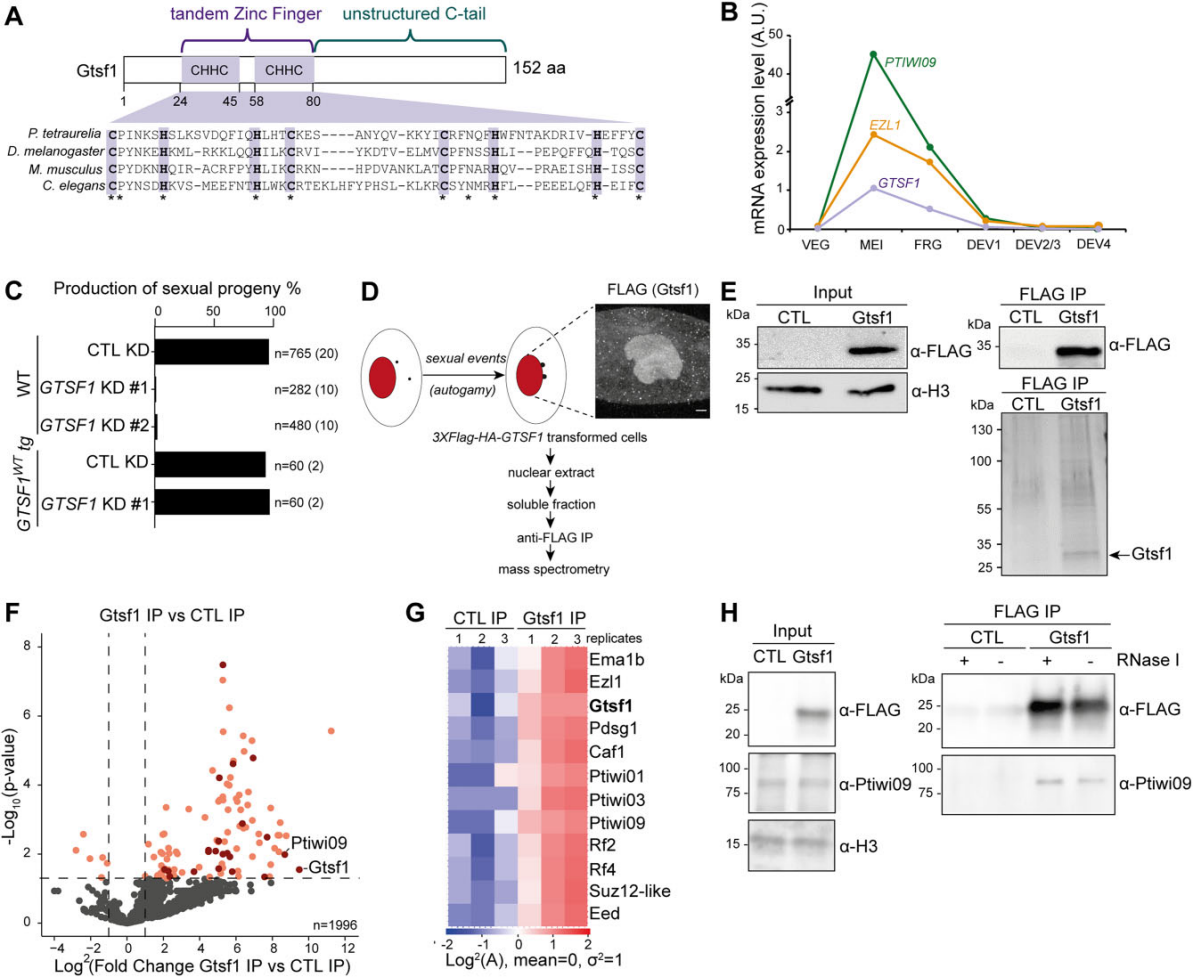
Gtsf1 is an essential nuclear protein that interacts with Ptiwi09. (**A**) Schematic representation of Gtsf1-predicted protein domains and alignment of the N-terminal portion of Gtsf1 from *P. tetraurelia* (PTET.51.1.G0490019), *Drosophila melanogaster* (CG3893), *Mus musculus* (MGI:1 921 424) and *Caenorhabditis elegans* (CELE_T06A10.3) (from top to bottom). The conserved tandem CHHC zinc finger domain is highlighted in purple. (**B**) Messenger RNA (mRNA) expression levels of the genes encoding *GTSF1*, *EZL1* and *PTIWI09* at different developmental stages during autogamy in arbitrary units (45). (**C**) Production of sexual progeny of wild-type (WT) cells and cells expressing a 3XFLAG-HA-Gtsf1 (GTSF1-WT tg) fusion protein following *GTSF1* (with two independent fragments #1 and #2) or *ICL7* (CTL) RNAi-mediated silencing (KD). The total number of cells analyzed for each RNAi and the number of independent experiments (in parentheses) are indicated. (**D**) Schematic representation of Gtsf1 IP experiments. Anti-FLAG immunostaining of cells transformed with a *3XFLAG-HA-GTSF1* transgene, at the same time point as that used for preparation of nuclear extracts for IP (T = 0 h). Scale bar, 10 μm. (**E**) Top: Western blot analysis of nuclear extracts of *Paramecium* expressing a 3XFLAG-HA-Gtsf1 functional protein (Gtsf1) or 3XFLAG-HA (CTL) before (input) or after affinity purification (FLAG IP). Bottom: Silver-stained gel of pulled-down proteins. Predicted MW for 3XFLAG-HA-Gtsf1: 23.6 kDa. (**F**) Volcano plot of the quantitative label-free mass-spectrometry analysis of 3xFLAG-HA-Gtsf1 affinity purification. Three replicates were analyzed for each condition. Significantly enriched proteins in Gtsf1 IP over control IP (statistical *t*-test, the fold change is greater than 2, the total number of peptides is greater than 2 and the *P*-value is less than 0.05) that are encoded by developmental genes with an early expression peak are highlighted, and include Gtsf1 and Ptiwi09. (**G**) Heatmap of abundance of the 12 top protein hits in Gtsf1 IP over control IP. Log_2_ of abundance is transformed and standardized with a mean of 0 and variance of 1. Only the proteins encoded by developmental genes with an early expression peak were considered. (**H**) Western blot analysis of nuclear extracts of *Paramecium* expressing a 3XFLAG-HA-Gtsf1 functional protein (Gtsf1) or not (CTL) before (input) or after affinity purification (FLAG IP). The affinity purification experiment is performed in presence (+) or absence (−) of an RNase I treatment (see Supplementary Figure S2). Anti-FLAG, anti-Ptiwi09 and anti-H3 antibodies are used for detection.

For the 3xFLAG-HA-Gtsf1 IP (Figure 2 and Supplementary Table S2), *Paramecium* nuclear protein extracts were performed as previously described (Frapporti *et al.*, 2019). 10^6^ autogamous cells (T = ∼0 h) were lysed with a Potter-Elvehjem homogenizer in three volumes of lysis buffer [10 mM Tris (pH = 6.8), 10 mM MgCl_2_, 0.2% Nonidet *P*-40, 1 mM PMSF, 4 mM benzamidine, 1× Complete EDTA-free Protease Inhibitor Cocktail tablets (Roche)]. The nuclei-containing pellet was collected by centrifugation and washed with the addition of 2.5 volumes of washing solution [0.25 M sucrose, 10 mM MgCl_2_, 10 mM Tris (pH = 7.4), 1 mM PMSF, 4 mM benzamidine, 1× Complete EDTA-free Protease Inhibitor Cocktail tablets (Roche)]. The pellet was incubated in one volume of nuclear extraction buffer 2× [100 mM Hepes (pH = 7.8), 100 mM KCl, 30 mM NaCl, 0.2 mM EDTA, 20% glycerol, 2 mM Dithiothreitol (DTT), 0.02% Nonidet *P*-40, 2 mM PMSF, 2× Complete EDTA-free Protease Inhibitor Cocktail tablets (Roche)] for 1 h at 4°C. The salt-extractable fraction at 15 mM NaCl was recovered following centrifugation for 3 min at 10 000 *g* at 4°C. Nuclear extracts were incubated overnight at 4°C with 150 μl anti-FLAG M2 magnetic beads (M8823, Sigma) that were pre-washed with 1 ml of the buffer [20 mM Tris (pH = 8), 0.1 mM EDTA, 10% glycerol, 150 mM NaCl, 0.01% Nonidet *P*-40], named TEGN thereafter. Beads were washed five times with TEGN buffer and eluted with 3xFLAG peptide (F4799, Sigma–Aldrich) (45 μl) and the same volume of TEGN buffer at 4°C for 5 h. RNase I treatment in anti-FLAG Gtsf1 IP was performed as described in (16) except for the use of 2 mM Ribonucleoside Vanadyl Complex (VRC)(NEB) instead of 4 mM benzamidine. After incubation of half of the beads with 200 U of RNase I (Thermo Fisher Scientific) for 5 h at 4°C, beads were washed three times with TEGN buffer and boiled in Laemmli sample buffer.

### FLAG-Ptiwi09 IP for ubiquitylation analysis

A total of 200 ml (3600 cells/ml) of *Paramecium* cells transformed with the 3x*FLAG-PTIWI09* transgene at T = 0 h were lysed in 3 ml cold lysis buffer [50 mM Tris (pH = 8), 300 mM NaCl, 1% Triton X-100, 0.01% NP-40, 10% glycerol, 5 mM EDTA, 25 mM N-Ethylmaleimide (Sigma), 1× Inhibitor cocktail cOmplete™ ULTRA Tablets EDTA free (Roche), 2mM PMSF] using a Potter-Elvehjem homogenizer until all nuclei were destroyed. The lysate was incubated for 1 h at 4°C and centrifuged for 30 min at 18 400 *g*. The supernatant was incubated overnight at 4°C with 50 μl of anti-FLAG M2 magnetic beads (M8823, Sigma). The beads were washed five times with freshly prepared lysis buffer, two times 10 min with high salt buffer [10 mM Tris (pH = 7.4), 1 M NaCl], two times 10 min with 10 mM Tris (pH = 7.4), 1 M urea and finally two times for 10 min in 10 mM Tris (pH = 7.4), 150 mM NaCl. The beads were boiled for 10 min at 95°C in 100 μl 1× LDS Sample Buffer (Invitrogen).

### Western blot and silver staining

For western blot, electrophoresis and blotting were carried out according to standard procedures. Samples were run on 10% Tris-Glycine Bio-Rad gels. Blotting was performed overnight using a nitrocellulose (GE10600002, Merck) or polyvinylidene fluoride (PVDF) membrane (Bio-Rad). FLAG (1:1000 or 1:4000) (MAI-91878, Thermo Fisher Scientific or F1804, Sigma), *Paramecium* H3 (1:5000) (20), histone H3.3 (PA5-23288, Invitrogen) (1:4000), α-tubulin (1:5000) (sc-8035, Santa Cruz) or TEU435 (1:4000) (35) were used for primary antibodies. Secondary horseradish peroxidase-conjugated anti-mouse or anti-rabbit IgG antibodies (Promega) were used at 1:2500 or 1:8000 dilution followed by detection by ECL.

To detect ubiquitin, samples were run on NuPage 3–8% Tris-Acetate Gel at 4°C 90V. Blotting was performed overnight using a PVDF membrane (F1804, Sigma). The membrane was boiled for 30 min in water and incubated for 1 h in 5% skim milk in Tris-buffered saline pH 7.4 (TBS) then for 2 h in primary antibody (1:2000 ubiquitin monoclonal antibody 13–1600 Invitrogen) and for 1 h at room temperature in secondary antibody (1:4000 anti-Mouse-HRP, Promega).

In order to detect the endogenous Ptiwi09 protein, polyclonal rabbit antibodies were raised to the QLANTEIVNKKAGTK peptide sequence of the *Paramecium* Ptiwi09 protein with Eurogentec (Seraing, Belgium) and were purified by antigen affinity purification. Antibody α-Ptiwi09-433 specificity was tested on western blot (1:2000) using whole cell extracts extracted as previously described (36). Cell pellets were aliquoted, frozen in liquid nitrogen and kept at −80°C. Cell pellets were lysed by the addition of an equal volume of boiling 5% sodium dodecyl sulfate (SDS) containing 1× Complete EDTA-free Protease Inhibitor Cocktail tablets (Roche) and boiled for 5 min. Protein containing supernatants were collected after centrifugation at 16 000 g for 5 min at 4°C and boiled in Laemmli sample buffer. Electrophoresis was carried out according to standard procedures using 4–20% Tris-Glycine Bio-Rad gels. Blotting was performed in 25 mM phosphate buffer for 1.5 h at 0.5 ampere using a Amersham Protran Premium 0.45 μm NC membrane.

Silver staining was carried out with SilverQuest (Invitrogen LC6070) according to the manufacturer's instructions.

### Mass spectrometry

#### Sample preparation

For Ptiwi09 IP, the agarose beads were analyzed at the Mass Spectrometry Laboratory at the Institute of Biochemistry and Biophysics PAS. At first, cysteines were reduced by 1 h incubation with 20 mM Tris(2-carboxyethyl)phosphine at 60°C followed by 10 min incubation at room temperature with 50 mM methyl methanethiosulfonate. Digestion was performed overnight at 37°C with 1 μg of trypsin (Promega). The tryptic digestion was stopped by lowering the pH of the reaction below pH 4 by adding extraction buffer (0.1% Trifluoroacetic Acid (TFA) 2% acetonitrile (ACN)). The agarose beads were separated from solution by centrifugation. The resulting peptide mixtures were applied to RP-18 pre-column (Waters, Milford, MA, USA) using water containing 0.1% formic acid (FA) as a mobile phase and then transferred to a nano-High Performance Liquid Chromatography (HPLC) RP-18 column (internal diameter 75 μM, Waters, Milford MA) using acetonitrile (ACN) gradient (0 – 35% ACN in 160 min) in the presence of 0.1% FA at a flow rate of 250 nl/min. The column outlet was coupled directly to the ion source of an Orbitrap Elite mass spectrometer (Thermo Electron Corp., San Jose, CA, USA) working in the regime of data-dependent MS to MS/MS switch. A blank run ensuring absence of cross-contamination from previous samples preceded each analysis.

For Gtsf1 IP, gel plugs were discolored using a solution of ACN/NH4HCO3 50 mM (50/50) for 15 min with agitation, reduced with a 10-mM DTT solution for 45 min at 56°C, then alkylated using a 55-mM iodoacetamide (IAA) solution for 45 min at room temperature. After a washing and dehydration step, proteins in the plugs were digested overnight with trypsin (Promega) at 37°C in a 25-mM NH4HCO3 buffer (0.2 μg trypsin in 20 μl). The digested peptides were loaded and desalted on evotips provided by Evosep (Odense, Denmark) according to the manufacturer’s instructions before liquid chromatography tandem mass spectrometry (LC-MS/MS) analysis at the Mass Spectrometry Facility at Institut Jacques Monod. The samples were analyzed on a timsTOF Pro 2 mass spectrometer (Bruker Daltonics, Bremen, Germany) coupled to an Evosep one system (Evosep, Odense, Denmark) operating with the 30SPD method developed by the manufacturer. Briefly, the method is based on a 44-min gradient and a total cycle time of 48 min with a C18 analytical column (0.15 × 150 mm, 1.9 μm beads, ref EV-1106) equilibrated at 40°C and operated at a flow rate of 500 nl/min. H_2_O/0.1% FA was used as solvent A and ACN/0.1% FA as solvent B. The timsTOF Pro 2 was operated in parallel accumulation serial fragmentation (PASEF) mode1 over a 1.3 s cycle time. Mass spectra for MS and MS/MS scans were recorded between 100 and 1700 *m/z*. Ion mobility was set to 0.75–1.25 V·s/cm2 over a ramp time of 180 ms. Data-dependent acquisition was performed using six Parallel Accumulation Serial Fragmentation (PASEF) MS/MS scans per cycle with a near 100% duty cycle. Low *m/z* and singly charged ions were excluded from PASEF precursor selection by applying a filter in the *m/z* and ion mobility space. The dynamic exclusion was activated and set to 0.8 min, a target value of 16 000 was specified with an intensity threshold of 1000. Collisional energy was ramped stepwise as a function of ion mobility.

### Data analysis

MS raw files were processed using PEAKS Online X (build 1.8, Bioinformatics Solutions Inc.). Data were searched against the ParameciumDB database (*P. tetraurelia* protein annotation v2.0, download 2021_10, total entries 40 460). Parent mass tolerance was set to 10 ppm for Ptiwi09 IP and 25 ppm for Gtsf1 IP, the fragment mass tolerance to 0.05 Da. Specific tryptic cleavage was selected and a maximum of two missed cleavages was authorized. For identification, the following post-translational modifications were included: oxidation (M) and deamidation (NQ) as variables and beta-methylthiolation (C) as fixed. Identifications were filtered based on a 1% false discovery rate threshold at PSM level. Label free quantification was performed using the PEAKS Online X quantification module, allowing a mass tolerance of 20 ppm and a retention time shift tolerance of 1 min for match between runs for Ptiwi09 IPs and a Collision Cross Section (CCS) error tolerance of 0.05 and a retention time shift tolerance in autodetect for match between runs for Gtsf1 IP. Protein abundance was inferred using the top N peptide method and TIC was used for normalization. Multivariate statistics on proteins were performed using Qlucore Omics Explorer 3.8 (Qlucore AB, Lund, SWEDEN). A positive threshold value of 1 was specified to enable a log_2_ transformation of abundance data for normalization i.e. all abundance data values below the threshold will be replaced by 1 before transformation. The transformed data were finally used for statistical analysis i.e. evaluation of differentially present proteins between two groups using a Student’s bilateral *t*-test and assuming equal variance between groups. A *P*-value better than 0.05 was used to filter differential candidates.

### Immunofluorescence and quantification

As described in (20), cells were fixed for 30 min in solution I [10 mM EGTA, 25 mM HEPES, 2 mM MgCl_2_, 60 mM PIPES (pH = 6.9), PHEM 1X; 1% formaldehyde, 2.5% Triton X-100, 4% sucrose], and for 10 min in solution II (PHEM 1X, 4% formaldehyde, 1.2% Triton X-100, 4% sucrose). Following blocking in 3% bovine serum albumin-supplemented Tris buffered saline-Tween 20 0.1% for 10 min, fixed cells were incubated overnight at room temperature under agitation with primary antibodies as follows: rabbit anti-H3K9me3 (1:200) (20), rabbit anti-H3K27me3 (1:1000) (20) and mouse anti-FLAG (1:200) (MAI-91878, Thermo Fisher Scientific). Cells were labeled with Alexa Fluor 568-conjugated goat anti-rabbit IgG, Alexa Fluor 488-conjugated goat anti-rabbit IgG or Alexa Fluor 568-conjugated goat anti-mouse IgG at 1:500 for 1 h, stained with 1 μg/ml Hoechst for 5–10 min and finally mounted in Citifluor AF2 glycerol solution.

The pre-extraction procedure was performed as follows: cells were permeabilized for 3.5 min in PHEM 1X with 1% Triton X100 then fixed in PHEM 1X with 2% formaldehyde for 10 min. This procedure was compared with cells fixed for 10 min in PHEM 1X with 2% formaldehyde then permeabilized for 15 min in PHEM 1X with 1% Triton X100. The permeabilization and fixation steps were done with gentle agitation. Cells were then incubated with anti-FLAG antibodies and processed as described above. Images were acquired using an Eclipse TE2000-E inverted microscope equipped with a Nikon C1 confocal laser scanning head and a CFI Plan Apochromat 60× Oil objective. Z-series were performed with Z-steps of 1 μm. To measure protein levels within the maternal MAC, with or without pre-extraction, quantification was performed using ImageJ on cells at the ‘skein’ stage, when the maternal MAC begins to fragment. Convolution was applied to the DAPI channel using a gaussian filter with a full width at half-maximum of 2 pixels. Image segmentation was then conducted using the Otsu’s method for automatic thresholding. Background spots as well as holes smaller than 2 pixels wide in the nucleus were excluded from consideration. The accuracy of the mask was visually checked to ensure that the selected object corresponded to the maternal MAC at the appropriate stage. This generated mask was used to calculate the sum of FLAG fluorescence intensities across all Z-stacks (FLAG fluorescence intensity) for each nucleus. This value was divided by the nucleus volume obtained by segmentation (in voxels). For each condition at least 30 nuclei were quantified. Mann–Whitney statistical tests were performed with GraphPad Prism, and statistical details of the experiments can be found in the figure legends.

Other images were acquired using a Zeiss LSM 780 or 980 laser-scanning confocal microscope and a Plan-Apochromat 63×/1.40 oil DIC M27 or a Plan-Apochromat 40×/1.3 oil DIC objective. Z-series were performed with Z-steps of 0.35 μm. Quantification was performed as previously described (36) using ImageJ. The volume of the nucleus (in voxels) was estimated as follows: using the Hoechst channel, the top and bottom Z-stacks of the developing MAC were defined to estimate nucleus height in pixels. The equatorial Z-stack of the developing MAC was defined, and the corresponding developing MAC surface was measured in pixels. The estimated volume of the developing MAC was then calculated as the product of the obtained nucleus height by the median surface. For each Z-stack of the developing MAC, the H3K9me3 or H3K27me3 fluorescence intensity was measured and corrected using the ImageJ ‘subtract background’ tool. The sum of the corrected H3K9me3, H3K27me3 or FLAG fluorescence intensities for all the Z-stacks, which corresponds to the total H3K9me3, H3K27me3 or FLAG fluorescence intensity, was divided by the estimated volume to obtain the H3K9me3, H3K27me3 or fluorescence intensity per voxel in each nucleus. For each condition at least 30 nuclei were quantified. Mann–Whitney statistical tests were performed with GraphPad Prism. All of the statistical details of experiments can be found in the figure legends.

### Chromatin immunoprecipitation

Chromatin immunoprecipitation (ChIP) experiments were performed with H3K9me3 or H3K27me3 antibodies as previously described (20). For spike-in, 10 μg of sonicated *D. melanogaster* chromatin prepared from ovaries as described in (37) was added to each sonicated *Paramecium* chromatin sample before IP. From ChIP-enriched samples and inputs, DNA was extracted with phenol, precipitated with glycogen in sodium acetate and ethanol and resuspended in deionized distilled water. Enrichment compared to input was analyzed by quantitative PCR (qPCR) and values were normalized to a *D. melanogaster* locus known to be enriched in H3K9me3 (42AB): region 1 (chr2R: 6 449 409–6 449 518). The RPL32 locus was used as a negative control. qPCR was performed using LightCycler^®^ 480 SYBR Green I Master (catalog number 04 707 516 001, Roche) on the Light Cycler 480 system (Roche). qPCR amplification was done with primers listed in Supplementary Table S3.

### DNA extraction and sequencing

DNA for deep-sequencing was isolated from post-autogamous cells (T = ∼50 h or T = 60 h) as previously described (11). Briefly, cells were lysed with a Potter-Elvehjem homogenizer in lysis buffer [0.25 M sucrose, 10 mM MgCl_2_, 10 mM Tris (pH = 6.8), 0.2% Nonidet *P*-40]. The nuclei-containing pellet was washed with washing buffer [0.25 M sucrose, 10 mM MgCl_2_, 10 mM Tris (pH = 7.4)], loaded on top of a 3-ml sucrose layer [2.1 M sucrose, 10 mM MgCl_2_, 10 mM Tris (pH = 7.4)] and centrifuged in a swinging rotor for 1 h at 210 000 *g*. The nuclear pellet was collected and diluted in 200 μl of washing buffer prior to addition of three volumes of proteinase K buffer (0.5 M EDTA pH 9, 1% N-lauryl sarcosine sodium, 1% SDS, 1 mg/ml proteinase K). Following overnight incubation at 55°C, genomic DNA was purified and treated with RNase A.

DNA from sorted new MACs subjected to deep sequencing was obtained using fluorescence-activated nuclear sorting at T = 25 h after the onset of autogamy upon *GTSF1* or CTL KD, as described in (38). Briefly, nuclei were collected from a 500 ml culture of autogamous cells at T = 25 h and subjected to flow cytometry sorting. A total of 60 000–100 000 new MACs were sorted based upon their Pgml1 labelling (38), and used for subsequent genomic extraction using QIAamp DNA micro kit (Qiagen). A microscope slide with 500 sorted nuclei was prepared in parallel to check for nuclear integrity and ploidy and anti-Pgm antibodies [Pgm 2659 GP (39)] were used.

Genomic DNA libraries were prepared either with the Westburg NGS DNA Library prep kit, or with the KAPA DNA HyperPrep (Kapa Biosciences, KK8504) and adapters Integrated DNA Technologies (IDT) for Illumina TruSeq DNA UD Indexes (Illumina 20 022 370), according to the manufacturer recommendations. The quality of the final libraries was assessed with an Agilent Bioanalyzer, using an Agilent High Sensitivity DNA Kit. Library concentration was determined by qPCR using the Kapa Library Quantification kit (Kapa Biosciences, KK4824), according to manufacturer’s instructions. Libraries were pooled in equimolar proportions and sequenced using paired-end 2 × 75 pb runs, on an Illumina NextSeq500 instrument, using NextSeq 500 High Output 150 cycles kit for the samples, or using paired-end 2 × 100 bp runs on an Illumina NovaSeq 6000 instrument, using NovaSeq 6000 S1 Reagent Kit (200 cycles) (Illumina, 20 028 318) with the 0.5% addition of control library Phix (Illumina, FC-110–3001). Sequencing metrics are available in Supplementary Table S4.

### RNA extraction and sequencing

Total RNA samples were extracted as previously described (20) from 200–400 ml of culture at 500 cells/ml for vegetative cells or at 2000–4000 cells/ml at different time-points during autogamy. Briefly, cells were centrifuged and flash-frozen in liquid nitrogen prior to TRIzol treatment, modified by the addition of glass beads for the initial lysis step. Alternatively, long and short RNA molecules were isolated from *Paramecium* cells using RNAzol® RT (Molecular Research Center). Total RNA quality was assessed with an Agilent Bioanalyzer 2100, using Agilent RNA 6000 pico kit. Directional polyA RNA-Seq libraries were constructed using the TruSeq Stranded mRNA library prep kit, following the manufacturer’s instructions. The quality of the final libraries was assessed with an Agilent Bioanalyzer, using a High Sensitivity DNA Kit. Libraries were pooled in equimolar proportions and sequenced using paired-end 2 × 75 pb runs, on an Illumina NextSeq500 instrument, using NextSeq 500 High Output 150 cycles kit.

Small RNAs of 15–35 nt were purified from total RNA on a 15% Tris-Borate-EDTA (TBE)/urea gel. Alternatively, small RNAs were first enriched using RNAzol® RT (Molecular Research Center) from frozen cells then purified on gels. Small RNA libraries were constructed using the NEBNext Small RNA kit or TruSeq Small RNA Library Prep (Illumina, RS-200–0012, RS-200–0036) according to the manufacturer’s recommendations. The quality of the final libraries was assessed with an Agilent Bioanalyzer, using an Agilent High Sensitivity DNA Kit. Libraries were pooled in equimolar proportions and sequenced using a single read 75 bp run on an Illumina NextSeq500 instrument, using NextSeq 500 High Output 75 cycles kit, or paired-end 100 bp run on an Illumina NovaSeq 6000 instrument, using NovaSeq 6000 S1 Reagent Kit (Illumina, 20 028 318). Sequencing metrics are available in Supplementary Table S4.

### Small RNA isolation and IP

A total of 200 ml of cells were collected at T = 0 and T = 25 h after the onset of autogamy and RNA was isolated using RNazol RT; 5 μg of small RNA fraction was run on 15% TBE/urea gel using Owl™ S4S sequencing gel system (Thermo Scientific) and small RNAs of 20–35 nt were gel-purified for library construction (input). FLAG-Ptiwi09 IP was performed as described in (8). For 3xFLAG-HA-Gtsf1 IP, the same protocol was used with a modified lysis buffer [50 mM Tris HCL (pH = 8), 75 mM NaCl, 10 mM EDTA, 1 mM DTT, 2 mM Ribonucleoside Vanadyl Complex (VRC), 0,3% Triton X-100, 10% glycerol, 1× Complete EDTA-free Protease Inhibitor Cocktail tablets (Roche)] to preserve the interaction with the Ptiwi09 protein. After IP, RNA was extracted with phenol:chlorophorm, precipitated, dried, resuspended and denatured in Gel Loading Buffer II (AM8546G Thermo Fisher Scientific), and run on a 15% TBE/urea gel. The gel was stained with SYBR Gold Nucleic Acid Gel Stain. Small RNAs of 20–35 nt were gel-purified and used for library construction (IP).

### RT-PCR

A total of 5 μg of large RNA fraction isolated from frozen cells with RNAzol RT (MRC) was treated with TURBO^®^ DNase (Thermo Fischer Scientific), extracted with phenol pH 4.3 and then with chlorophorm, and precipitated. RNA concentration was estimated using Nanodrop (Thermo Fisher Scientific). A total of 1 μg of RNA was reversed-transcribed using RevertAid H Minus Reverse Transcriptase (Thermo Fischer Scientific) and random hexamer. PCR amplification was done with primers listed in Supplementary Table S3.

### Analysis of sequencing data

Sequencing data were demultiplexed using CASAVA (v1.8.2) and bcl2fastq2 (v2.18.12), then adapters were removed using cutadapt (v3.4). Reads were mapped using Bowtie2 (v2.2.9) on known contaminants (mitochondrial genomes, ribosomal DNA and bacterial genomes). The sequencing data were mapped on *P. tetraurelia* strain 51 MAC (ptetraurelia_mac_51.fa), MAC + IES (ptetraurelia_mac_51_with_ies.fa) and MIC (ptetraurelia_mic2.fa) reference genomes using Bowtie2 (v2.2.9 –local -X 500), Hisat2 (v2.1.0, –rna-strandness FR –min-intronlen 20 –max-intronlen100) or BWA (v0.7.15 -n 0) for DNA-sequencing (DNA-seq), mRNA-sequencing (mRNA-seq) or sRNA-sequencing (sRNA-seq) data, respectively. For genome browser screenshots, the sequencing coverage was normalized using deeptools (bamCoverage v3.2.1 –binSize 1 –normalizeUsing CPM). Gene annotation v2.0 (ptetraurelia_mac_51_annotation_v2.0.gff3), IES annotation v1 (internal_eliminated_sequence_PGM_ParTIES.pt_51.gff3) and TE annotation (ptetraurelia_mic2_TE_annotation_v1.0.gff3) were used in this study. All files are available from the ParameciumDB download section (https://paramecium.i2bc.paris-saclay.fr/download/Paramecium/tetraurelia/51/) (40). R (v4.0.4) packages were used to generate images (ggplot2 v3.3.5; ComplexHeatmap v2.6.2; GenomicRanges v1.42; rtracklayer v1.50, seqinr v4.2–8, circlize v0.4.13, FactoMineR v2.4). Sequencing metrics are available in Supplementary Table S4.

IES retention was evaluated using ParTIES (MIRET module v1.05 default parameters).

The reads per kilo base per million mapped reads (RPKM) coverage on TEs or genes was calculated using the reads counts, determined by htseq-count (v0.11.2, –mode = intersection-nonempty; for RNA-seq the option –stranded = yes has been used) on filtered BAM files (samtools v1.3.1 -q 30) then normalized by the number of mapped reads on the MIC genome.

The small RNA (sRNA) reads (20–30 nt) were successively mapped on the MAC, MAC + IES and MIC genomes to attribute them to a specific genome compartment: MAC-destined sequence (MAC), IES or OES. The 23 nt siRNA reads that map to the RNAi targets were not considered. Normalized read counts were calculated using the number sequenced reads with a G + C content < 50%, compatible with a *Paramecium* G + C genomic content (∼27%).

## Results

### Identification of Gtsf1, a Ptiwi09-interacting protein in the maternal MAC

To uncover candidate proteins involved in scnRNA selection, we identified the protein partners of Ptiwi09 during the developmental stage at which scnRNA selection occurs. Whole cell extracts were prepared from *Paramecium* control cells and cells expressing a 3xFLAG-tagged Ptiwi09, at an early stage of the sexual process of autogamy, when the protein is present in the maternal MAC (Figure 1A and Supplementary Figure S1). IP of the FLAG tag, in two and four replicates for the control and Ptiwi09, respectively (see the ‘Materials and methods’ section), was followed by MS (Figure 1B and C). Statistical analyses revealed 283 differential proteins (out of 633 identified proteins) in the Ptiwi09 IP compared with control (fold change > 2; *P* value < 0.05; unique peptide > 2) (Figure 1C and Supplementary Table S2). We recovered Ptiwi09 as expected, and its paralog Ptiwi03, a previously reported partner of Ptiwi09 in the new developing MAC (Figure 1C) (16). We also identified PRC2 core components (Ezl1, Caf1, Eed) and PRC2 cofactors (Rf2 and Rf4) (Figure 1C and Supplementary Table S2). These proteins were recently shown to be involved in the scnRNA selection process (4,21), and the Rf4 cofactor was further shown to physically interact with Ptiwi09 in the new developing MAC (16). Another Ptiwi09 interactor we identified was an uncharacterized, small protein (18 kDa) referred to as Gtsf1 (Figures 1C and 2A, and Supplementary Table S2). Interestingly, it had not been identified among the protein partners of Ptiwi09 when purified from the new developing MAC at a later stage of autogamy (16).

Like its counterparts in other organisms [*D. melanogaster* (41), *M. musculus* (42) and *C. elegans* (43)], *Paramecium* Gtsf1 contains a double CHHC zinc finger domain at the N-terminus and an unstructured C-terminus (Figure 2A). It is important to note that this Gtsf1 protein is distinct from two U11-48K-like CHHC zinc finger proteins previously reported as Gtsf1 homologs (44). RNA-seq expression data (45) shows that the *GTSF1* gene displays a developmental expression profile similar to that of *PTIWI09* and *EZL1* (Figure 2B). To investigate Gtsf1 function, we performed KD experiments using RNAi during the sexual cycle of autogamy. KD of *GTSF1* leads to lethality of the sexual progeny, whereas KD of a control non-essential gene leads to survival of the post-autogamous progeny (Figure 2C and Supplementary Table S1). Thus, *GTSF1* is an essential gene during the sexual cycle.

To confirm the interaction between Gtsf1 and Ptiwi09, we performed reciprocal IPs using a functional tagged 3XFLAG-HA-Gtsf1 protein capable of rescuing the lethality caused by *GTSF1* KD (Figure 2C). To immunoprecipitate Gtsf1, we prepared nuclear extracts from control *Paramecium* cells and cells expressing the functional fusion protein. As expected, immunofluorescence experiments confirmed that Gtsf1, like Ptiwi09 (4,7), localizes in the maternal macronucleus during meiosis (Figure 2D). IP of the FLAG tag in triplicates for control and Gtsf1 (Figure 2E and Supplementary Figure S1) was followed by MS (Figure 2F and G). MS analyses revealed that Gtsf1 (bait) and the scnRNA-binding protein Ptiwi09 were significantly enriched in Gtsf1 IPs compared with controls, confirming the interaction between Gtsf1 and Ptiwi09 (Figure 2F). Statistical analyses revealed 95 differential proteins (95 of 1996 identified proteins) in the Gtsf1 IP compared with control (fold change > 2; *P*-value < 0.05; unique peptides > 2) (Figure 2F and Supplementary Table S2). The top differential proteins encoded by developmental genes with an early expression peak, similar to Gtsf1 and Ptiwi09, included the Ptiwi09 paralogs Ptiwi01 and Ptiwi03 (7) and the Ptiwi09-associated protein Ema1b (16), a putative RNA helicase (33) (Figure 2F and G and Supplementary Table S2). Its *Tetrahymena* counterpart (Ema1p) was reported to be required for scnRNA selection (27). We also identified the PRC2 core components Ezl1, Caf1, Suz12-like and Eed, and PRC2 cofactors Rf2 and Rf4, confirming the interaction between Gtsf1, Ptiwi09 and PRC2. In addition, we identified Pdsg1, a protein previously reported to be involved in IES elimination and scnRNA selection (46).

To assess whether the interaction between Gtsf1 and Ptiwi09 might involve RNA, we examined Gtsf1 IP after control or RNase I treatment, in two independent biological replicates (Figure 2H and Supplementary Figures S1 and S2). Using custom anti-Ptiwi09 antibodies (see the ‘Materials and methods’ section; Supplementary Figure S2), we detected Ptiwi09 in the Gtsf1 IP but not in the control IP, as expected. After treatment with RNase I, Ptiwi09 was still found in the Gtsf1 IP, consistent with an RNA-independent interaction between Gtsf1 and Ptiwi09.

**Figure 3.**
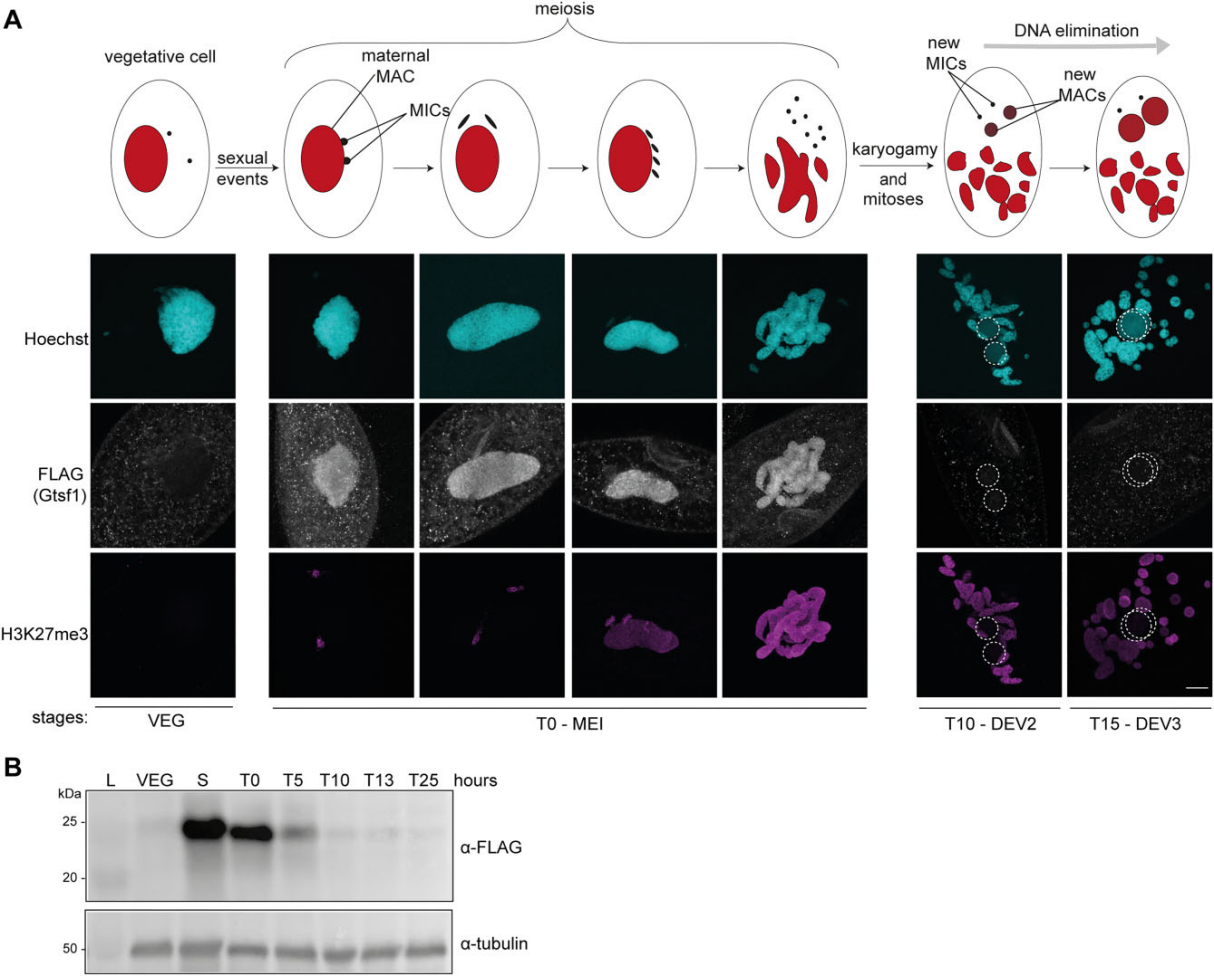
Gtsf1 is localized in the maternal MAC. (**A**) Co-immunostaining with FLAG and H3K27me3 antibodies on cells expressing 3XFLAG-HA-Gtsf1 during vegetative life (VEG) and at different stages of the *Paramecium* sexual cycle (autogamy). T = 0 h (the onset of autogamy) is defined as 50% of cells are autogamous (25% have a fragmented maternal MAC), as evaluated by cytological observation. Stages are MEI (T = 0 h), DEV2 (T = 10 h), DEV3 (T = 15 h), as previously described (45). Representative images are displayed. Overlay of Z-projections of magnified views of Hoechst staining (top), FLAG- (middle) and H3K27me3- (bottom) specific antibodies are presented. The new developping MACs (dashed white circles) are round and display a faint and smooth Hoechst signal. The other Hoechst-stained nuclei are the fragments of the maternal MAC and the MICs. Note that meiotic MICs (T0-MEI) are labeled with H3K27me3. Scale bar, 10 μm. (**B**) Western blot analysis of whole cell extracts at different time points (VEG, vegetative; S, starved; T = 0; 5; 10; 13; 25 h at the onset of autogamy, see Supplementary Figure S1 for cytology) with FLAG antibodies to detect 3XFLAG-HA-Gtsf1 and tubulin antibodies for normalization.

To gain further insight into the role of Gtsf1, we examined its subcellular localization. Co-immunofluorescence experiments were performed with *Paramecium* cells expressing the functional FLAG-tagged Gtsf1 under its endogenous regulatory regions at different stages of sexual events using FLAG antibodies and specific H3K27me3 antibodies (20) (Figure 3A). The FLAG signal is detected in the MAC at the onset of meiosis I, as determined by H3K27me3 labeling of the MICs (22). Gtsf1 appears in the maternal MAC before this nucleus is fragmented, and before it accumulates detectable H3K27me3. Gtsf1 persists until the formation of the new developing MAC, when the signal vanishes from the fragmented MAC. Western blot analysis with FLAG antibodies confirmed this early pattern of expression (Figure 3B). Consistent with H3K27me3 being detected later than Gtsf1 in the maternal MAC, Gtsf1 localization was shown to be independent of Ezl1 (Supplementary Figure S2). Thus, Gtsf1 is the first protein identified so far that localizes in the maternal MAC during MIC meiosis, and appears absent from the new MAC.

### Gtsf1 is required for efficient DNA elimination and TE silencing

**Figure 4.**
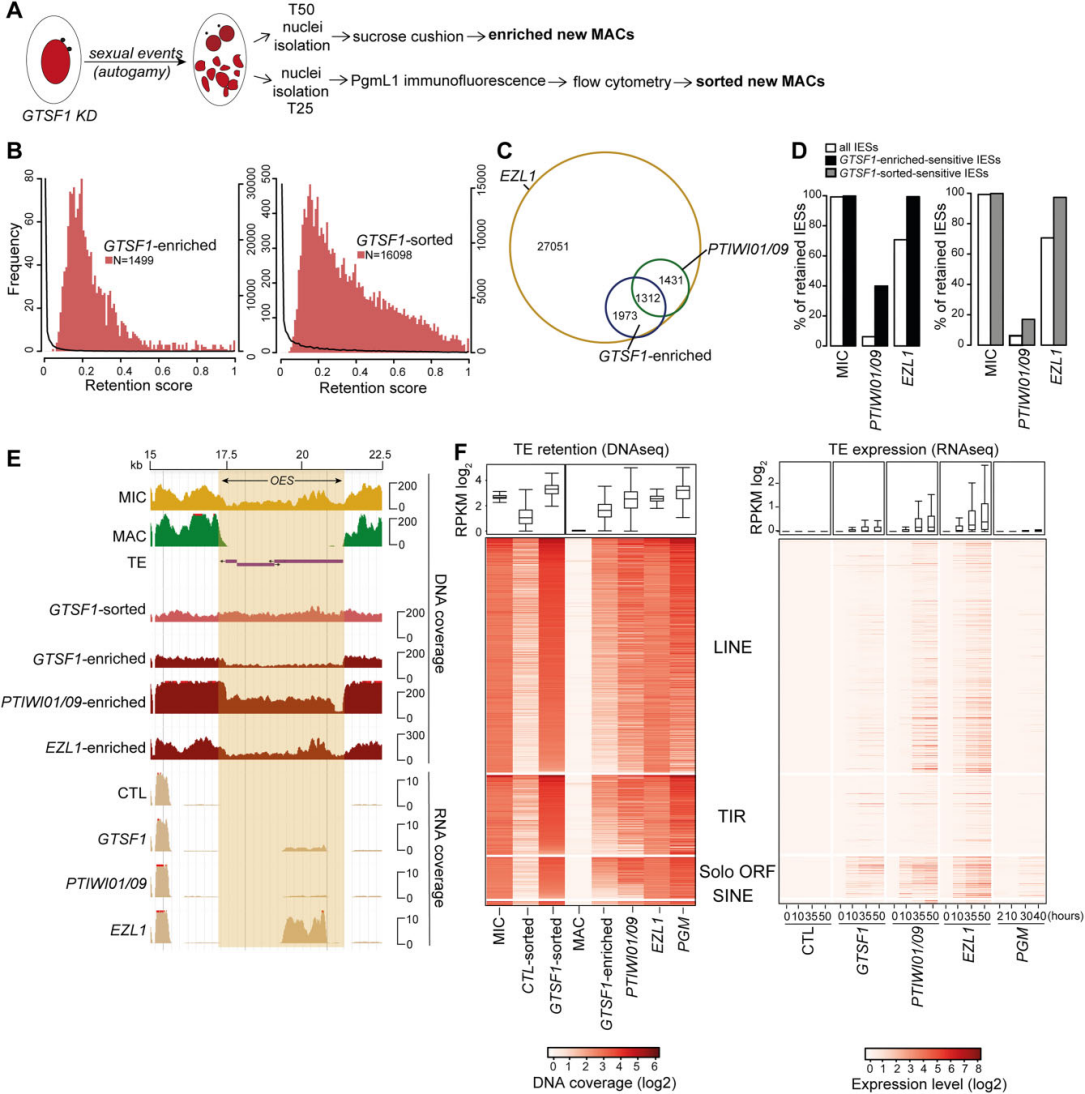
Gtsf1 is required for efficient DNA elimination and TE silencing. (**A**) Schematic representation of the experimental design to extract genomic DNA from GTSF1-silenced cells. (**B**) Histograms of IES retention scores upon *GTSF1* KD. The significantly retained IESs in *GTSF1* KD are represented by the red histograms (scale on the left), while the global distribution for all IESs retained in *GTSF1* KD is represented by the black curve (scale on the right). (**C**) Venn diagram of significantly retained IESs upon different KDs [enriched new MACs in all conditions, two GTSF1-enriched replicates (R1 and R2) are combined; Supplementary Figure S3]. (**D**) Histogram of the percentage of retained IESs [all, GTSF1-enriched, GTSF1-sorted] in MIC, *EZL1* or *PTIWI01/09* KDs. (**E**) Representative genomic region depicting DNA coverage and RNA coverage (T = 50 h) in *ND7* (CTL), *GTSF1*, *EZL1* and *PTIWI01/09* KD (NODE_36 852_length_47 919_cov_44.754879 between 15 and 22.5 kb). (**F**) Left panel: Heatmaps of TE normalized DNA coverage in each KD. Right panel: Heatmap of RNA expression levels at different time points during development in each KD. TE copies are ordered by the mean DNA coverage of GTSF1-enriched and GTSF1-sorted in each family (LINE *n* = 770, TIR *n* = 261, SOLO ORF *n* = 136 and SINE *n* = 13). The coverage distribution (RPKM log_2_) for all TE copies is shown as a boxplot. The box shows the first and third quartiles. The median is displayed as a horizontal line. The outliers are not drawn and the whiskers run from the minimum to the maximum value.

Given that Gtsf1 is a Ptiwi09 partner and that depletion of the Ptiwi01/09 proteins impairs DNA elimination (7,8,16), we examined the impact of *GTSF1* KD on DNA elimination. Sequencing of genomic DNA extracted from two preparations enriched for new MACs upon depletion of Gtsf1 (*GTSF1-enriched*) (Figure 4A and Supplementary Figure S3) indicated that 3.3%–6.6% of IESs are significantly retained (1499 and 3001, respectively, *P*-value < 0.05) (Figure 4B and Supplementary Figure S3). The smaller subset was almost totally included in the larger one, with 3285 affected IESs in both samples (Figure 4C). This number is likely underestimated because of contamination of the sample preparation by fragments of the maternal MAC. For this reason, we performed sequencing of genomic DNA extracted from new MACs sorted by flow cytometry from cells depleted for *GTSF1* (*GTSF1-sorted*) (Figure 4A and Supplementary Figure S4). We found that 35.7% of IESs, a larger subset indeed, are significantly retained (16 098, *P*-value < 0.05) (Figure 4B and Supplementary Figure S3). Previous sequencing studies of genomic DNA extracted from preparations enriched for new MACs showed that a small subset of IESs depend on Ptiwi01/09 (6.1%) (8,16), all of which require Ezl1 for their excision (22). We found that 3.3%–6.6% of the IESs that are significantly retained in *GTSF1* KD are essentially all (97.3%) included in the *EZL1*-dependent subset and that they are enriched in scnRNA-dependent IESs (Figure 4C and D and Supplementary Figure S3). We also noted that Gtsf1-dependent IESs are enriched for longer IESs (Supplementary Figure S3).

To analyze the effects on the elimination of MIC-limited sequences other than IESs, we examined TEs and found that all four of the major distinct TE families (14) are retained upon *GTSF1* KD, similarly to *EZL1* and *PTIWI01/09* KDs (Figure 4E and F and Supplementary Figure S3). Given that TE transcript levels are increased upon depletion of Ptiwi01/09 and of the PRC2 components (16), and to determine whether this was also the case upon depletion of Gtsf1, we performed RNA-seq at the same developmental stages upon *GTSF1* KD (T = 0, T = 10, T = 35 and T = 50 h after the onset of autogamy) (Figure 4E and F and Supplementary Figure S1). A total of 5% of all annotated TE copies become expressed (>1 RPKM) during MAC development (T = 50 h) upon *GTSF1* KD (Figure 4E and F). TE de-silencing in Gtsf1-depleted cells is weaker than in Ptiwi01/09- (14%) or Ezl1- depleted cells (25%) but is specific, as no TE expression is detected in cells depleted for the elimination machinery (Pgm), when all DNA elimination events are blocked (Figure 4F).

In addition to TEs, the expression of a few thousand developmental genes is deregulated upon *EZL1* KD (20), raising the question of whether Gtsf1 might contribute to their regulation as well. We therefore used our RNAseq dataset to evaluate the impact of *GTSF1* KD on the expression of genes whose expression is either upregulated (*N* = 1505) or downregulated (*N* = 870) upon *EZL1* KD. We found that these genes are indeed deregulated upon *GTSF1* KD (Supplementary Figure S3). Focusing on the 628 developmental genes that are up-regulated upon KD of the elimination machinery (Pgm, Ku80 and Xrcc4) (47), we found that depletion of either Gtsf1 or Ezl1 also affects the expression of these genes (Supplementary Figure S3). Thus, Gtsf1 depletion, as that of other proteins essential for DNA elimination, results in the deregulation of a subset of developmental genes, and this is likely a response to defective IES excision in the new MAC.

### Gtsf1 depletion affects H3K9me3 and H3K27me3 levels and localization

**Figure 5.**
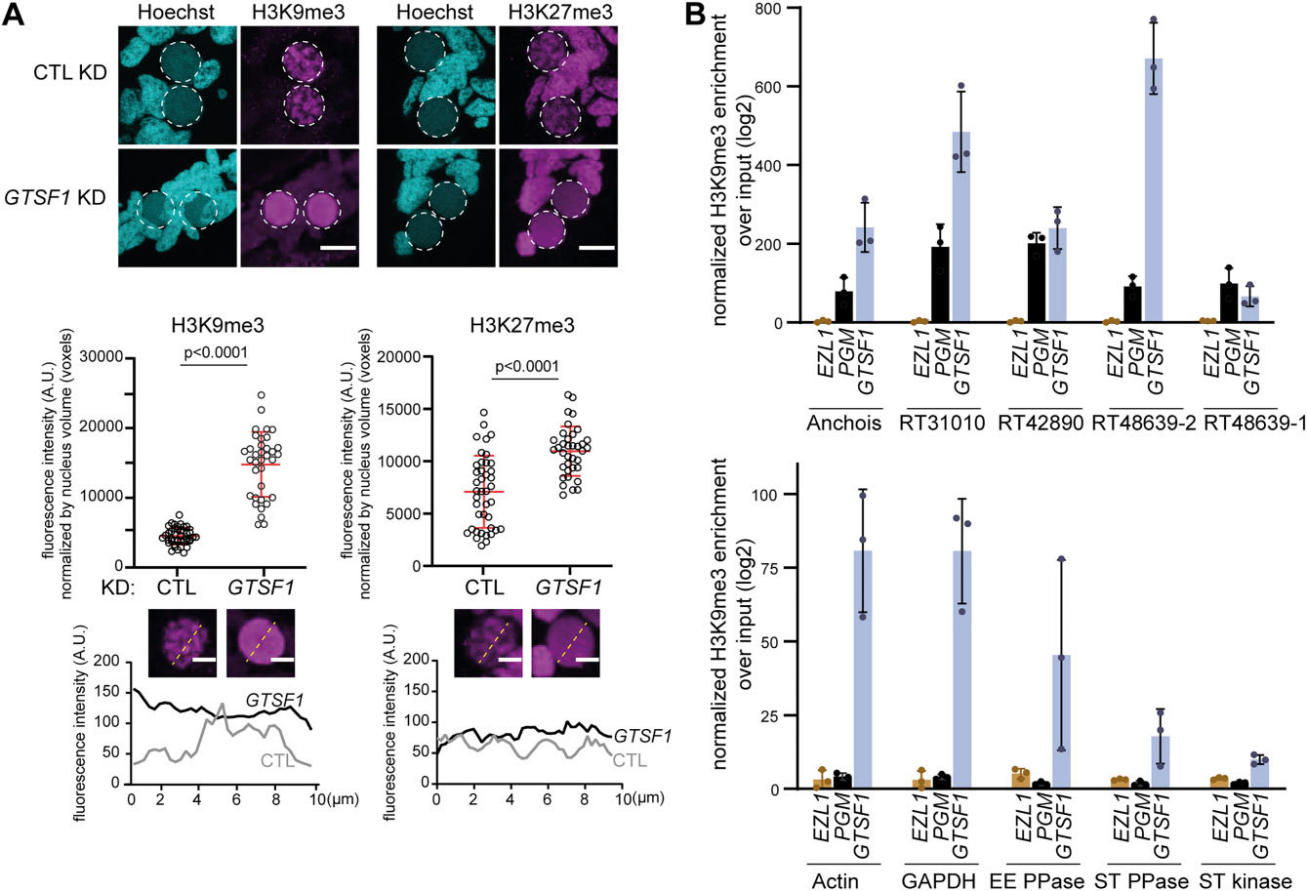
Gtsf1 depletion affects H3K9me3 and H3K27me3 levels and localization. (**A**) H3K9me3 or H3K27me3 antibodies are used for immunostaining of WT cells at T = 15 h after the onset of autogamy in *ICL7* (CTL) or *GTSF1* KD. Dashed white circles indicate the new developing MACs. The other Hoechst-stained nuclei are the fragments of maternal MAC and the MICs. Note that H3K9me3 staining is visible in the fragments of the maternal MAC upon *GTSF1* KD, while it is not in *CTL* KD. Scale bar, 10 μm. Middle: Quantification of H3K9me3 and H3K27me3 fluorescence signal in the new MAC (see the ‘Materials and methods’ section) (see Supplementary Figure S5). Number of nuclei > 30 in each condition. Bars correspond to mean ± standard deviation (SD). Mann–Whitney statistical tests. Bottom: Quantification of H3K9me3 and H3K27me3 fluorescence signal along the line crossing the nucleus. Scale bar, 2 μm. (**B**) Barplots of normalized H3K9me3 enrichment over input (log_2_) for TE copies (*ANCHOIS*, *RT31010*, *RT42890*, *RT48639-2*, *RT48639-1*) and for genes (*ACTIN*, *GAPDH*, *EE PPASE*, *ST PPASE*, *ST KINASE*) determined by ChIP-qPCR upon *EZL1*, *PGM* and *GTSF1* KD (three replicates for each KD). Bars correspond to mean ± SD.

We examined whether depletion of Gtsf1 had an effect on accumulation of H3K9 and H3K27 trimethylation during autogamy using immunofluorescence. H3K9me3 and H3K27me3 accumulate in the new MAC in control conditions (Figure 5A), as previously described (20,22). In contrast, H3K9me3 and H3K27me3 no longer accumulate in the new MAC if any of the core components of PRC2 is absent, and their levels are diminished in the absence of Ptiwi01/09 (16). Interestingly, however, H3K9me3 and H3K27me3 could still be detected in the new MAC upon depletion of Gtsf1 (Figure 5A). Quantification of H3K9me3 and H3K27me3 fluorescence in the new developing MACs indicated that H3K9me3 and H3K27me3 accumulation is in fact significantly increased upon Gtsf1 depletion (Figure 5A and Supplementary Figure S5). Additionally, we found that a functional tagged Ezl1, the catalytic subunit of PRC2 (20), accumulates in the maternal MAC, where it is normally barely detected, upon Gtsf1 depletion (Supplementary Figure S5).

In control conditions, H3K9me3 and H3K27me3 signals in new developing MACs display a diffuse pattern that gradually forms nuclear foci, as previously reported (Figure 5A) (22). In Gtsf1-depleted cells, however, the H3K9me3 and H3K27me3 signal remains diffuse as development proceeds and foci are not detected (Figure 5A). Thus, despite the fact it is localized in the maternal MAC, Gtsf1 controls both the levels of Ezl1-mediated H3K9me3 and H3K27me3 and the clustering of these marks into nuclear foci in the new developing MACs, a function reminiscent of that reported for downstream effectors of the DNA elimination pathway (22,36).

To determine whether H3K9me3 and H3K27me3 are correctly targeted to TEs, we performed ChIP experiments (T = 50 h) upon *GTSF1* KD, and upon *PGM* KD as a positive control. ChIP-qPCR analysis showed that TEs are enriched for both H3K27me3 and H3K9me3 in *PGM* KD conditions, while genes are not enriched for these marks (Supplementary Figure S5), as previously reported (20). By contrast, H3K9me3 and H3K27me3 enrichment are reduced for the TEs we analyzed upon *GTSF1* KD (Supplementary Figure S5). Thus, though Gtsf1 is confined to the maternal MAC, it appears to be required for proper targeting of H3K9me3 and H3K27me3 to TEs in the new developing MACs.

The decreased enrichment of PRC2-deposited marks we detect by ChIP-qPCR could reflect a straightforward reduction of H3K9me3 and H3K27me3 at TEs or, alternatively, given our cytological observations, a uniformly elevated distribution of these marks throughout the genome at the expense of specific TE enrichment. To distinguish between these scenarios, we developed a spike-in ChIP-qPCR procedure using exogenous *Drosophila* chromatin for normalization (see the ‘Materials and methods’ section). Spike-in H3K9me3 ChIP was performed in *PGM*, *EZL1* and *GTSF1* RNAi. ChIP-qPCR analysis showed that the five TEs tested are enriched for H3K9me3 upon *PGM* KD, while no enrichment can be detected upon *EZL1* KD (Figure 5B), as expected (20). We found that TEs are enriched for H3K9me3 upon *GTSF1* KD and that the enrichment is higher upon *GTSF1* KD than upon *PGM* KD for some TEs (Figure 5B). Interestingly, while no enrichment is detected upon *EZL1* or *PGM* KD on genes (Figure 5B) as expected (20), we found H3K9me3 enrichment on genes upon *GTSF1* KD (Figure 5B), and this does not correlate with reduction in gene expression levels (Supplementary Figure S3). Altogether, these data support the idea that the abundant PRC2-mediated histone marks that accumulate in Gtsf1-depleted cells are more equally distributed on the genome and less focused on TEs than the marks in control cells.

### Gtsf1 is required for scnRNA selection

**Figure 6.**
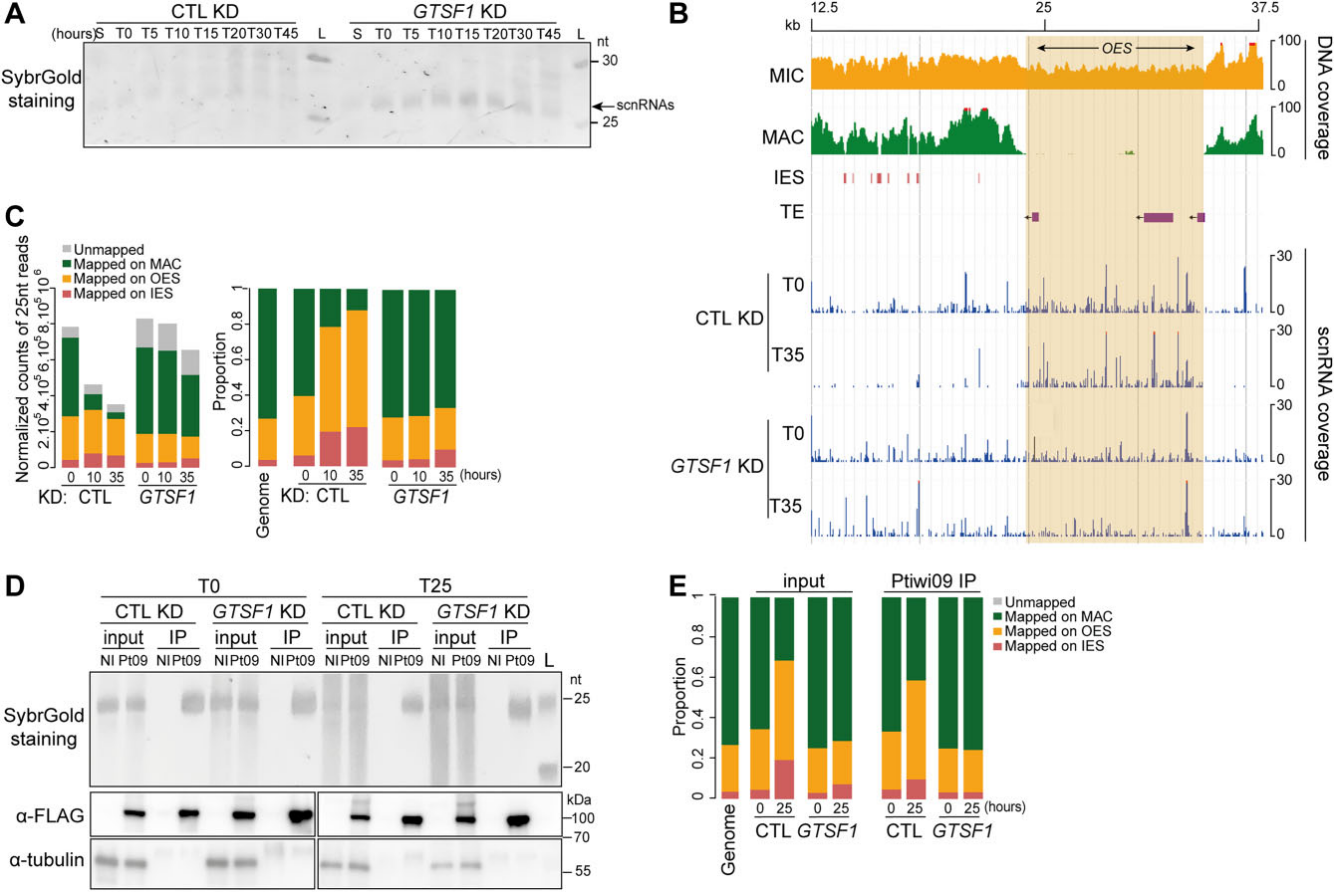
Gtsf1 is required for scnRNA selection. (**A**) Gel electrophoresis of sRNAs from *ND7* (CTL) and *GTSF1* KD cells. Total RNA samples corresponding to different time points (S = starvation, T = 0, 5, 10, 20, 30 and 45 h after the onset of autogamy) were run on a denaturing 15% polyacrylamide-urea gel. After electrophoresis, the gel was stained with SybrGold. L: DNA low molecular weight marker (USB Corporation). The 25-nt scnRNAs are indicated. (**B**) Representative genomic region depicting 25-nt scnRNA normalized coverage in *ICL7* (CTL) and *GTSF1* KD (NODE_1273_length_39 901_cov_28.471216 between 12.5 and 37.5 kb). (**C**) Analysis of 25-nt scnRNA populations in *ICL7* (CTL) and *GTSF1* KD at different times points (T = 0, 10 and 35 h after the onset of autogamy). Bar plots show the normalized counts of 25-nt reads for each sample that map the MAC genome, IESs or MIC-limited sequences (left) and the proportion of 25-nt reads for each category (right). Genome: proportion of each category (MAC, OES, IES) in the MIC genome (see Supplementary Figure S6). (**D**) Upper panel: Gel electrophoresis of sRNAs before (input) or after (Ptiwi09 IP) IP of cells expressing *3X-FLAG-PTIWI09* and of non-injected cells (NI) at two different time points (T = 0 and 25 h after the onset of autogamy) in *ICL7* (CTL) and *GTSF1* KD. Total RNA samples were run on a denaturing 15% polyacrylamide-urea gel. After electrophoresis, the gel was stained with SYBR Gold. L: molecular weight marker (GeneRuler Ultra Low Range DNA ladder). Middle and lower panels: Western blot analysis of whole cell extracts from the same samples as the upper panel. Ptiwi09 detection was performed using FLAG antibodies and tubulin antibodies were used for normalization. (**E**) Sequencing analysis of scnRNA populations before (input) or after (Ptiwi09 IP) IP at T = 0 and 25 h after the onset of autogamy in *ICL7* (CTL) or *GTSF1* KD [same samples as in panel (D)]. Genome: proportion of each category (MAC, OES, IES) in the MIC genome (see Supplementary Figure S6).

Our data show that Gtsf1 affects H3K9me3 and H3K27me3 targeting and DNA elimination in the new developing MAC, yet it is present in the maternal MAC. This suggests that Gtsf1 plays a role in the maternal MAC during scnRNA selection. To investigate this possibility, we first evaluated steady-state scnRNA levels after depletion of Gtsf1 compared with control knockdown at eight different time points during autogamy (Supplementary Figure S1). This indicated that loss of Gtsf1 leads to increased total accumulation of scnRNAs (Figure 6A). In contrast, loss of Ptiwi01/09 leads to destablization of scnRNAs (7).

We extended the scnRNA analysis by sequencing total small RNAs in the 20- to 30-nt range from Gtsf1-depleted cells at several time points in two independent time-course experiments (from T = 0 to T = 35 h after the onset of autogamy) (Supplementary Figures S1 and S6). In Gtsf1-depleted cells, the 25-nt scnRNAs are produced from the whole MIC genome at the beginning of the sexual cycle (T = 0 h), as in control RNAi conditions, consistent with previous work (4) (Figure 6B and C). This can be clearly observed when examining the mapping of 25-nt scnRNAs across an individual genomic region (Figure 6B), where MAC sequences, as well as MIC sequences (IES and OES), are covered. We therefore conclude that Gtsf1 is not involved in the process of scnRNA biogenesis.

As autogamy proceeds (T = 10 and T = 35 h), the proportion of 25-nt scnRNAs mapping to OES or IES over scnRNAs mapping to MAC sequences increases in control conditions (Figure 6B and C). At T = 10 h, scnRNAs mostly corresponded to MIC sequences (Figure 6C). However, MIC-specific scnRNAs (OES + IES) are not enriched over MAC-specific scnRNAs in Gtsf1-depleted cells, indicating an absence of MIC-specific scnRNA selection (Figure 6B and C, and Supplementary Figure S6). As illustrated for an individual genomic region, scnRNAs still cover IESs and the entire OES region, which comprises annotated TEs, at T = 35 h (Figure 6B). These patterns are in stark contrast to those observed upon loss of Ptiwi01/09 (Supplementary Figure S6) and instead resemble those observed upon loss of PRC2-Ezl1 components (4,21). Importantly, because we still detect maternal MAC ncRNA production in the *GTSF1* knockdown experiments, the lack of scnRNA selection cannot be explained by a lack of maternal MAC ncRNA transcription (Supplementary Figure S7).

To determine whether the scnRNAs corresponding to the entire germline genome that accumulate in Gtsf1*-*depleted cells are still loaded onto Ptiwi09, we performed Ptiwi09 IP followed by small RNA isolation (8) (see see the ‘Materials and methods’ section) upon *GTSF1* and control KD at two different time points (T = 0 and T = 25 h) (Figure 6D and Supplementary Figure S1). Sequencing of small RNAs revealed the presence of 25-nt scnRNAs in control, as expected (8), and in Gtsf1-depleted cells at both time points (Supplementary Figure S6). While the proportion of MIC-scnRNAs increases in the control at T = 35 h as expected, this is not the case upon Gtsf1 depletion, as the same proportions of MIC- and MAC- scnRNAs are detected in the IP at both time points, mirroring what is seen in the input (Figure 6E and Supplementary Figure S6). This indicates that the non-selected scnRNAs that accumulate in Gtsf1-depleted cells are bound to Ptiwi09.

### Gtsf1 controls Ptiwi09 protein levels and ubiquitylation, and trimming of bound scnRNAs

**Figure 7.**
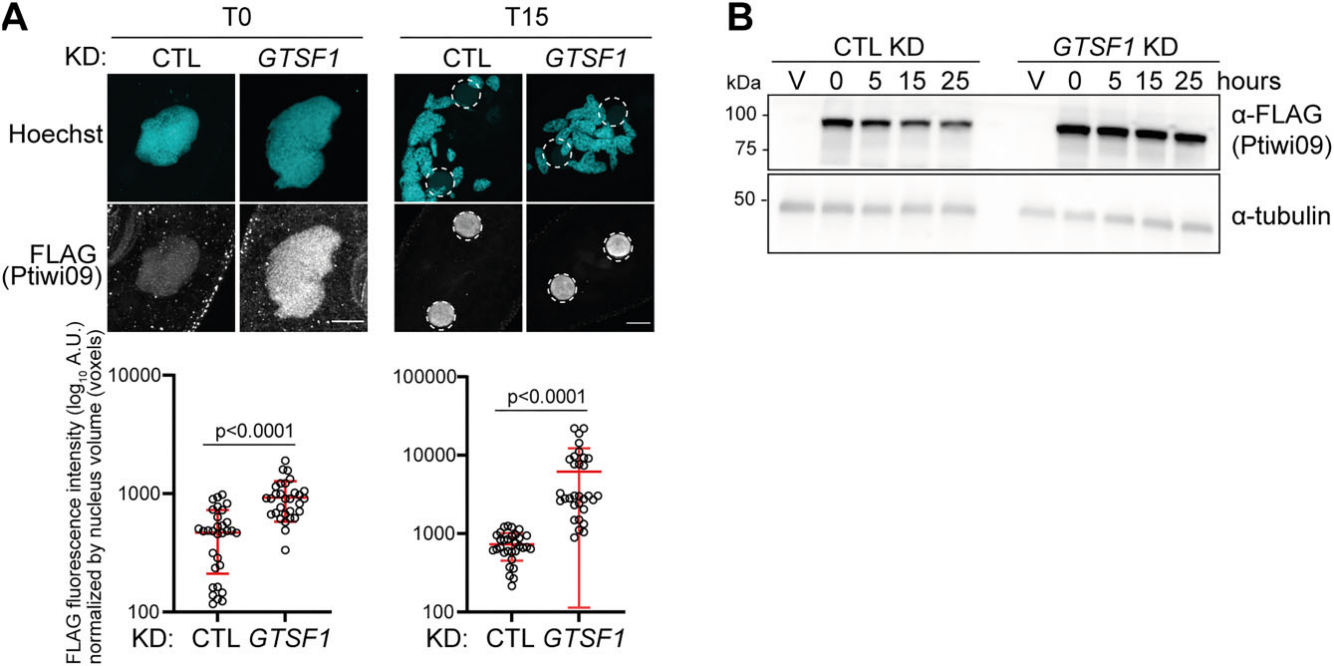
Gtsf1 controls Ptiwi09 protein levels. (**A**) Anti-FLAG immunostaining of cells transformed with a *3xFLAG-HA-PTIWI09* transgene at T = 0 and T = 15 h after the onset of autogamy in *ICL7* (CTL) or *GTSF1* KD. Dashed white circles indicates the new developing MACs. The other Hoechst-stained nuclei are the fragments of maternal MAC. Scale bar, 10 μm. Quantification of FLAG fluorescence signal in the nuclei (see the ‘Materials and methods’ section). Number of nuclei > 30 in each condition. Bars correspond to mean ± SD. Mann–Whitney statistical tests. (**B**) Western blot analysis of whole cell extracts at different time points [Vegetative (V); T = 0, 5, 15, 25 h after the onset of autogamy] in *ND7* (CTL), *GTSF1* and *PTIWI01/09* KDs with FLAG antibodies to detect 3XFLAG-Ptiwi09 and tubulin antibodies for normalization.

To determine whether impairment of scnRNA selection could be explained by defects in the localization of scnRNAs, we analyzed the localization of the scnRNA-binding protein Ptiwi09 with a FLAG*-*tagged *PTIWI09* transgene (16). In control cells, the Ptiwi09 fusion protein is detected in the maternal MAC during MIC meiosis, then localized in the developing MAC (Figure 7A), as previously reported (7). In cells lacking Gtsf1, the subcellular localization of Ptiwi09 proteins was unaffected (Figure 7A). Thus, the lack of MIC-specific scnRNA selection in the absence of Gtsf1 cannot be explained by the mis-localization of Ptiwi09-bound scnRNAs.

However, the intensity of the 3xFLAG-tagged-Ptiwi09 signal appeared increased in Gtsf1-depleted cells. Therefore, we quantified the fluorescence intensity (see the ‘Materials and methods’ section) and found a significant increase of nuclear Ptiwi09 levels in the maternal MAC compared to control conditions (Figure 7A). Western blot analysis from whole cell extracts confirmed that the steady-state levels of the Ptiwi09 protein increase in Gtsf1-depleted cells (Figure 7B and Supplementary Figures S1 and S2). Because the steady-state mRNA levels of *PTIWI01/09* are not impacted by *GTSF1* KD (Supplementary Figure S3), we conclude that Gtsf1 controls Ptiwi09 protein accumulation more directly.

**Figure 8.**
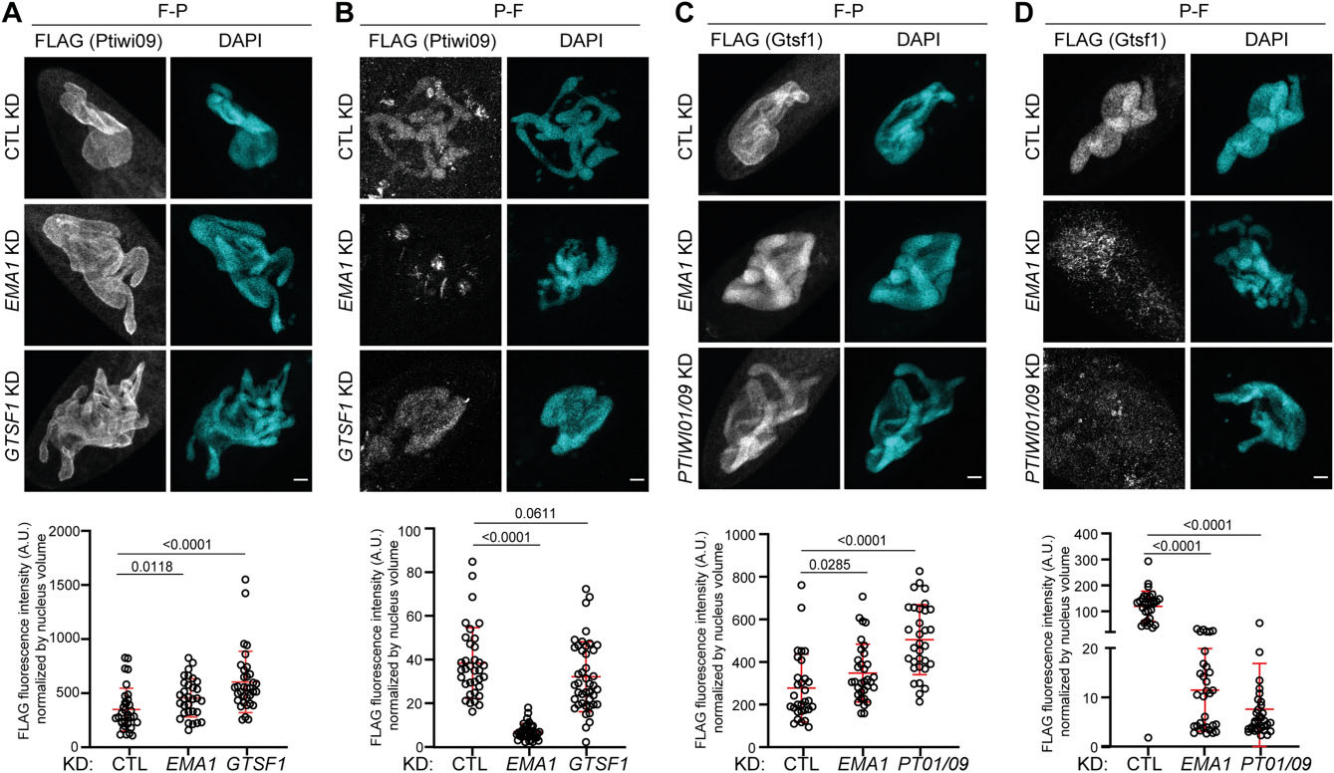
Ema1, but not Gtsf1, is required for Ptiwi09 binding to chromatin. (**A, B**) Anti-FLAG immunostaining of cells transformed with a *3xFLAG-PTIWI09* transgene in *ICL7* (CTL), *EMA1* or *GTSF1* KD at the onset of autogamy. (**C, D**) Anti-FLAG immunostaining of cells transformed with a *3XFLAG-HA-GTSF1* transgene in *ICL7* (CTL), *EMA1* or *PTIWI01/09*KD. Cells were either fixed then permeabilized [F-P, panels (A) and (C)], or permeabilized then fixed [P-F, panels (B) and (D)] in order to determine whether the protein is chromatin-bound. Scale bar, 10 μm. Quantification of FLAG fluorescence signal in the maternal MAC (see the ‘Materials and methods’ section). Number of nuclei > 30 in each condition. Bars correspond to mean ± SD. Mann–Whitney statistical tests. Bright fluorescence spots, outside the segmented nucleus, are visible in some FLAG images.

To assess whether Gtsf1 is associated with free-, or chromatin-bound- Ptiwi09, we included a Triton-mediated permeabilization step prior to cell fixation in the immunofluorescence protocol (see the ‘Materials and methods’ section). This pre-extraction procedure may affect the apparent localization of proteins that are not tightly bound to chromatin, which are washed out from the nucleus. Under these pre-extraction conditions, a fraction (approximately 10%) of the 3xFLAG-tagged-Ptiwi09 protein is not washed out, supporting the idea that Ptiwi09 is chromatin-bound (Figure 8). Upon *EMA1* KD under pre-extraction conditions, Ptiwi09 is no longer chromatin-bound (Figure 8). This is consistent with the idea that Ema1, which is required for scnRNA selection and DNA elimination (Supplementary Figure S8), plays a role in Ptiwi09 binding to chromatin, as previously reported for its *Tetrahymena* counterpart (27). In contrast, upon *GTSF1* KD under pre-extraction conditions, Ptiwi09 remains localized in the nucleus, indicating that Ptiwi09 binding to chromatin is independent of Gtsf1. Immunostaining of the 3xFLAG-HA-Gtsf1 protein indicates that the localization of Gtsf1 in the maternal MAC remains unaffected upon *EMA1* or *PTIWI01/09* KD. In contrast, immunostaining under pre-extraction conditions indicates that the fraction of the 3xFLAG-HA-Gtsf1 protein that remains bound to chromatin in control KD (Figure 8) is washed out from the nucleus upon *EMA1* KD or *PTIWI01/09* KD (Figure 8). This is consistent with the idea that Gtsf1 is associated with chromatin-bound Ptiwi09.

**Figure 9.**
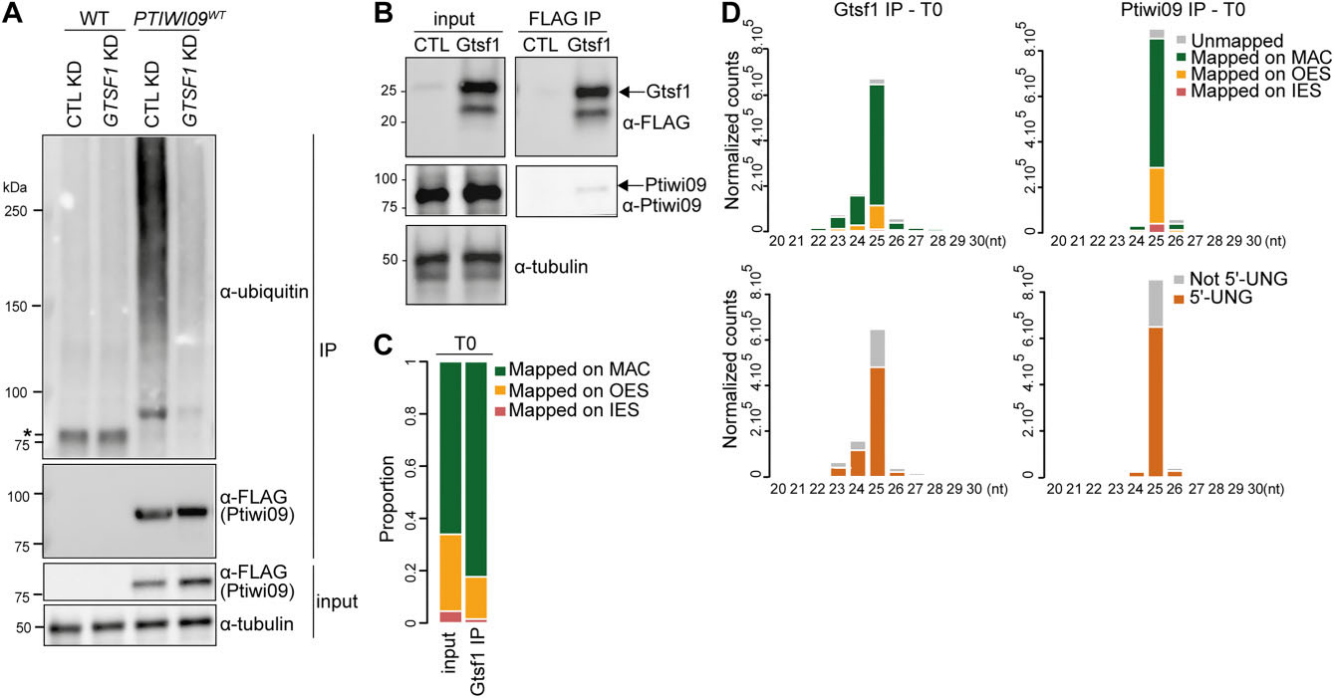
Gtsf1 controls Ptiwi09 ubiquitylation and trimming of bound-scnRNAs. (**A**) Western blot analysis of ubiquitylation of Ptiwi09 IP on WT and cells expressing *3XFLAG-PTIWI09* transgene (PTIWI09^WT^) at T = 0 h after the onset of autogamy upon *ICL7* (CTL) and *GTSF1* KD. Ptiwi09 detection was performed using FLAG antibodies before (input) and after (IP) Ptiwi09 IP. α-tubulin antibodies were used for normalization. *: crossreaction with beads. (**B**) Western blot analysis of 3XFLAG-HA-Gtsf1 RNA IP. (**C**) Analysis of 25-nt scnRNA populations of 3XFLAG-HA-Gtsf1 IP (Gtsf1 IP), at T = 0 h after the onset of autogamy. Bar plots show the proportion of 25-nt reads that map the MAC genome, IESs or OES sequences for the input and Gtsf1 IP (see Supplementary Figure S6). (**D**) Analysis of small RNA populations of 3XFLAG-HA-Gtsf1 (left) and 3XFLAG-Ptiwi09 (right, same sample as Figure 6E) IP experiments (at T = 0 h after the onset of autogamy). Up: Bar plots show the normalized counts of 20- to 30-nt reads for each sample that map the MAC genome, IESs or OES sequences. Down: Bar plots show the normalized counts of 20- to 30-nt reads for each sample that display a 5′-UNG end or not.

Given that the levels of the Ptiwi09 protein and the associated MAC-scnRNAs are increased in Gtsf1-depleted cells, we propose that Gtsf1 mediates the degradation of Ptiwi09/MAC-scnRNA complexes in the maternal MAC. This would explain the lack of MIC-scnRNA selection in the absence of Gtsf1. Given the general role of ubiquitin in triggering protein degradation (48) and the reported case of ubiquitylation-mediated degradation of Argonaute protein (49), we hypothesize that the ubiquitin pathway is involved in the degradation of Ptiwi09/MAC-scnRNA complexes. To test this possibility, we examined the levels of ubiquitylated proteins in Ptiwi09 IPs upon *GTSF1* and control KD, from cells collected at a stage when Ptiwi09 is detected in the maternal MAC (Figure 9A and Supplementary Figure S1). Anti-ubiquitin antibodies revealed that Ptiwi09 is ubiquitylated in a Gtsf1-dependent manner. Given that Ema1 interacts both with Ptiwi09 and Gtsf1, we wondered whether Ema1 might also control Ptiwi09 ubiquitylation. We therefore examined the levels of ubiquitylated proteins in Ptiwi09 IPs upon *EMA1* and control KD (Supplementary Figures S8 and S1) and found that Ptiwi09 is ubiquitylated in a Ema1-dependent manner. Altogether, we conclude that Gtsf1 and Ema1 control the ubiquitylation of Ptiwi09 during scnRNA selection.

To assess whether Ptiwi09 protein degradation involves the ubiquitin proteasome pathway, cells were treated with the proteasome inhibitor MG132 for a short period of time at the onset of autogamy (T = 0 h) (see the ‘Materials and methods’ section). Treatment with MG132 leads to increased levels of ubiquitylated proteins compared to control conditions, validating the efficacy of the proteasome inhibitor treatment (Supplementary Figure S9). The levels of the steady-state Ptiwi09 protein increase upon MG132 treatment compared to control (Supplementary Figure S9). Consistent with MG132 affecting cell cycle progression, we found that MG132 treatment impairs the exchange of gametic nuclei during conjugation and greatly reduces the production of viable sexual progeny (Supplementary Figure S9). Altogether, these are consistent with the idea that Ptiwi09 protein degradation involves the ubiquitin proteasome pathway.

Our data are consistent with a scenario in which Gtsf1 is able to discriminate the pool of Ptiwi09 proteins that is bound to MAC-scnRNAs from the pool that is bound to MIC-scnRNAs. To test the possibility that Gtsf1 interacts with the particular sub-population of Ptiwi09 proteins that is loaded with MAC-scnRNAs, we performed Gtsf1 IP followed by small RNA isolation at T = 0 time point. We used IP conditions (see the ‘Materials and methods’ section) that preserved the interaction between Gtsf1 and Ptiwi09, as indicated by western blot analysis (Figure 9B and Supplementary Figure S1). Sequencing of small RNAs revealed that the proportion of 25-nt MAC-scnRNAs detected in the IP, in two independent biological replicates, is increased in comparison with what is seen in the input (Figure 9C and Supplementary Figure S6). Yet MIC-scnRNAs are present in the IP, even though no scnRNAs matching IESs are detected. Thus, contrary to our expectations, 25-nt scnRNAs-bound Ptiwi09 that associates with Gtsf1 are not exclusively MAC-scnRNAs. We noticed the presence of 23- and 24-nt sRNA populations corresponding to MAC sequences in the Gtsf1 IP, that are not as abundant in the Ptiwi09 IP at the same time point (Figure 9D). The majority of 23-nt and 24-nt sRNAs detected in the Gtsf1 IP starts with 5′-UNG, like 25nt-scnRNAs (Figure 9D). The presence of the characteristic 5′-UNG signature of 25-nt scnRNAs (3) supports the idea that 24- and 23-nt sRNAs correspond to scnRNAs that have been shortened at their 3′ end by one or two nucleotides, respectively. Very interestingly, 3′ end trimming occurred while MAC-scnRNAs were bound to the Ptiwi09 protein. Thus, Ptiwi09-bound MAC-scnRNAs associated with Gtsf1 are caught in the process of being trimmed at their 3′ end, providing direct evidence of MAC-scnRNA degradation.

## Discussion

The nuclear *Paramecium* Gtsf1 homolog is an essential factor acting downstream of scnRNA biogenesis that is required for the selective degradation of a subpopulation of scnRNAs corresponding to MAC-destined sequences. As a result, scnRNA selection is defective in Gtsf1-depleted cells (Figure 6). Similar observations were recently reported in an independent preprint (50). Given its localization in the maternal MAC (Figure 3), the role of Gtsf1 in scnRNA selection reinforces the idea that the maternal MAC is where selective degradation of scnRNAs occurs.

Like its counterparts in other organisms (41,42,51–54), *Paramecium* Gtsf1 is required for Piwi-guided transcriptional silencing and repressive histone modifications at TE loci. In Gtsf1-depleted cells, TEs and a subset of IESs, which are enriched for long, scnRNA- and Ezl1-dependent sequences, are no longer eliminated in the new MAC (Figure 4 and Supplementary Figure S3). Some TEs are transcriptionally upregulated upon *GTSF1* KD, as previously reported for PRC2-Ezl1 components and *PTIWI01/09* KDs (16). Gtsf1 is thus a critical member of the DNA elimination pathway.

Other proteins involved in DNA elimination (Pdsg1, Ema1, Nowa1, PRC2 core complex and cofactors) are also necessary for scnRNA selection (4,13,21,27,46) (Supplementary Figure S8). However, in contrast to Gtsf1, these proteins localize both in the maternal MAC and in the new MAC. Given their dual roles and localization, it is difficult to unambiguously assign them a specific function in each nucleus. We previously reported that the methyltransferase activity of PRC2-Ezl1 is essential for DNA elimination (20), while its function in scnRNA selection does not involve its catalytic activity (4). Gtsf1 is the only reported case described so far of a protein that is present in the maternal MAC and yet has an effect on DNA elimination events that take place in the new MAC. Thus, our analysis of Gtsf1 provides a unique demonstration that defective scnRNA selection ultimately impairs DNA elimination.

When scnRNA selection occurs normally, Ptiwi09/MIC-scnRNA complexes represent the vast majority of Ptiwi09 complexes present in the new MAC (Figure 6 and Supplementary Figure S6) (8). In contrast, when scnRNA selection is defective in Gtsf1-depleted cells, we show that Ptiwi09 proteins present in the new MAC are loaded with both MAC- and MIC-scnRNAs (Figure 6 and Supplementary Figure S6). We also find that H3K9me3 and H3K27me3 become more abundant in the new MAC upon Gtsf1 depletion (Figure 5). Our ChIP data indicate that the Ptiwi09-scnRNA complexes guide the deposition of these repressive histone modifications along the genome with no specific enrichment on TEs (Figure 5 and Supplementary Figure S5). Surprisingly, H3K9me3-marked genes are not transcriptionally repressed (Supplementary Figure S3) and the more homogeneous distribution of repressive histone modifications along the genome does not appear to induce DNA elimination, suggesting that the mere presence of the repressive histone modifications is not sufficient to elicit the introduction of DNA double-strand breaks. We speculate that a threshold local concentration of histone modifications must be reached to trigger elimination of the modified chromatin. Another possibility is that the lack of differentially methylated regions, and thus of boundaries between eliminated regions and flanking regions, precludes the identification of the correct sites for introduction of DNA breaks by the elimination machinery. Finally, it might be the case that the Pgm endonuclease is limiting but still active such that a low level of elimination does in fact take place, but that it occurs randomly and homogeneously across the genome and is therefore not easily detectable when analyzing a pool of many cells.

Despite the fact that GTSF1 is conserved, its functions appear to have diversified. In both *Drosophila* and mouse, GTSF1 is also necessary for piRNA-dependent TE silencing; however, the mechanisms appear to be quite different. In *Drosophila*, DmGTSF1 localizes to the nucleus, and is required for transcriptional transposon silencing but not for piRNA biogenesis (41,52,53). In mouse, by contrast, GTSF1 localizes to both the cytoplasm and the nucleus, and is essential for secondary but not primary piRNA production (54). Mammalian GTSF1 appears to act as an auxiliary factor that potentiates the piRNA-directed RNA cleavage activities of PIWI proteins, transforming them into efficient endoribonucleases (55). In *C*. *elegans*, GTSF1 does not participate in the piRNA pathway, but is instead involved in the assembly of a complex that produces a specific class of endogenous small RNAs targeting genic transcripts (43). The function we have uncovered here for Gtsf1 in *Paramecium*, where it controls the selective degradation of scnRNAs, represents yet another example that underscores the divergent contributions of GTSF1 proteins to small RNA silencing pathways.

The somatic macronucleus, where Gtsf1 is localized in *Paramecium*, is devoid of TEs. Thus, the role of Gtsf1 in the control of TEs appears to be indirect. Like other Gtsf1 proteins, *Paramecium* Gtsf1 physically interacts with PIWI – the scnRNA binding protein Ptiwi09 (Figures 1–2) – but it has no effect on its nuclear localization (Figures 7 and 8), suggesting that Gtsf1 acts downstream of Ptiwi09. We show that Gtsf1 is associated with chromatin-bound Ptiwi09 and that the putative RNA helicase Ema1 is important for its tethering to chromatin (Figure 8). Given that PRC2 interacts with Gtsf1, Ptiwi09 and Ema1 (Figures 1 and 2), and that PRC2 is also required for scnRNA selection (4,21), the interplay between these different factors in the selection process is an important question for further investigation.

**Figure 10.**
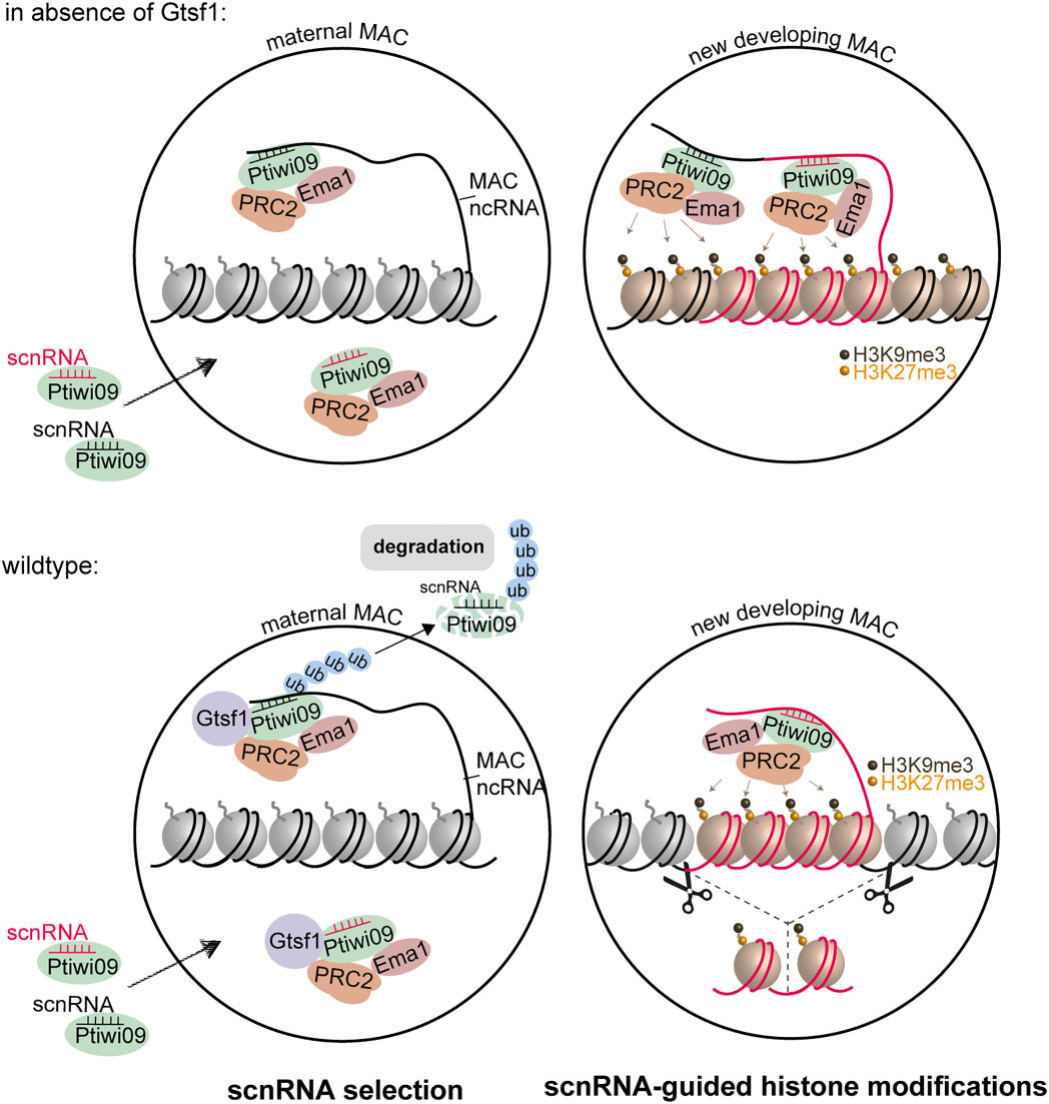
Model for the role of Gtsf1 in scnRNA selection. scnRNAs produced from the entire MIC genome during meiosis are bound to the Ptiwi09 protein and transported to the maternal macronucleus (MAC), where scnRNA selection occurs. The selective degradation of scnRNAs corresponding to MAC-destined sequences (black) results in the specific selection of the subpopulation corresponding to MIC-specific scnRNAs (in red). The Gtsf1 protein associates with all Ptiwi09 in the maternal MAC, irrespective of scnRNA sequence. We propose that the Gtsf1 protein when bound to Ptiwi09/MAC-scnRNA complexes engaged with target nascent noncoding transcripts triggers ubiquitylation of Ptiwi09, leading to degradation of Ptiwi09 and of its cognate scnRNAs (lower panel). scnRNAs corresponding to MIC-specific sequences (red), which by definition cannot pair with MAC transcripts, are retained and trigger DNA elimination in the new developing MAC.

We show that Gtsf1 controls the steady-state levels of the Ptiwi09 protein and of its bound MAC-scnRNAs, which both show increased accumulation in the nucleus upon *GTSF1* KD (Figure 7). We propose that Gtsf1 mediates the coordinated degradation of the subset of Ptiwi09 proteins that are loaded with MAC-scnRNAs as well as their bound MAC-scnRNAs (Figure 10). We envision that Gtsf1 interacts with chromatin-bound Ptiwi09 upstream of the selection process and binds all Ptiwi09 in the maternal MAC, irrespective of scnRNA sequence (Figures 7 and 9). According to our model (Figure 10), Ptiwi09/MAC-scnRNA complexes are engaged in pairing interactions with non-coding RNA transcribed from the somatic maternal MAC genome, while the Ptiwi09/MIC-scnRNA complexes are not, because of the lack of sequence complementarity between MIC-scnRNAs and MAC transcripts. When Gtsf1 binds Ptiwi09/MAC-scnRNA complexes engaged with target nascent noncoding transcripts, we propose that it triggers Ptiwi09 ubiquitylation and degradation and concomitant MAC-scnRNA degradation.

Our data lend support to the proposed mechanism for GTSF1 in metazoan whereby GTSF1 association with PIWI triggers a conformational change of PIWI upon target recognition, which can either lead to enhanced slicing activity or to changes in protein–protein interactions (29). We envision that, by analogy to what has been suggested for *M. musculus* and *Drosophila*, *Paramecium* Gtsf1 interacts with nascent transcripts from the maternal MAC to reinforce the association between Ptiwi09/MAC-scnRNA complexes and their targets.

How Ptiwi09 and its bound MAC-scnRNAs are degraded remains to be determined. Our data provide some hints that ubiquitylation mediated by Gtsf1 and Ema1 might be critical to this process. Indeed, we found that Ptiwi09 is ubiquitylated in a Ema1-, Gtsf1-dependent manner (Figure 9 and Supplementary Figure S8). One possible mechanism is that association with Gtsf1 causes the Ptiwi09 protein to undergo a conformational change upon scnRNA-target pairing. This could drive recruitment of E3 ubiquitin ligases, which in turn would trigger ubiquitylation and degradation of Ptiwi09, and scnRNA decay. Very interestingly, we also provide the first direct evidence of MAC-scnRNA degradation through 3′ end trimming while still bound to Ptiwi09 (Figure 9D). This suggests the existence of an active trimming mechanism involved in MAC-scnRNA degradation.

Target-directed degradation of scnRNAs during genome elimination in *Paramecium* exhibits striking similarities to recently reported instances of target-directed degradation of microRNAs in mammalian cells and *Drosophila* (56,57). miRNA degradation has been shown to rely on ubiquitylation and proteasomal degradation of the Argonaute protein (58,59). Loss of scnRNA degradation upon Gtsf1 disruption is accompanied by excess accumulation of Ptiwi09, suggesting that an analogous mechanism may be at play during *Paramecium* genome elimination. These highly diverged phenomena of small-RNA removal may thus share basic commonalities. Future work aiming at unraveling the underlying mechanisms of target-directed scnRNA degradation might reveal that target-directed small RNA degradation is an ancient process that is more widespread than previously thought.

## Supplementary Material

gkae1055_Supplemental_Files

## Data Availability

The datasets and computer code produced in this study are available in the following databases: DNA-Seq, RNA-Seq, sRNA-Seq data: European Nucleotide Archive (https://www.ebi.ac.uk/ena/browser/view/PRJEB65919 and PRJEB80873). The details can be found in Supplementary Table S3. Protein interaction AP-MS data: raw data PRIDE PXD045266 (http://www.ebi.ac.uk/pride/archive/projects/PXD045266) (Ptiwi09) and PRIDE PXD045214 (Gtsf1) (http://www.ebi.ac.uk/pride/archive/projects/PXD045214); processed data Zenodo (https://doi.org/10.5281/zenodo.13919430). Modeling computer scripts: zenodo (https://doi.org/10.5281/zenodo.13919430). The uncropped blots and the numerical values for each figure can be found in the Supplementary data.
