# Supplementary material for: The PIWI-interacting protein Gtsf1 controls the selective degradation of small RNAs in Paramecium
Source: Nucleic Acids Res. 2024 Nov 22;53(1):gkae1055. doi: 10.1093/nar/gkae1055 (PMC11724296; doi:10.1093/nar/gkae1055)
Supplement: gkae1055_Supplemental_Files [file gkae1055_supplemental_files.zip › Charmant_Gruchota_Supplementary Figures and Tables_revision NAR_20241028.pdf]

The PIWI-interacting protein Gtsf1 controls the selective degradation of small RNAs in *Paramecium*

Olivia Charmant*[1], Julita Gruchota*#[2], Olivier Arnaiz[3], Katarzyna P. Nowak[2], Nicolas Moisan[1], Coralie Zangarelli[3], Mireille Bétermier[3], Anna Anielska-Mazur[2], Véronique Legros[1], Guillaume Chevreux[1], Jacek K. Nowak[2], Sandra Duharcourt#[1]

[1] Université Paris Cité, CNRS, Institut Jacques Monod, F-75013 Paris, France
[2] Institute of Biochemistry and Biophysics Polish Academy of Sciences, Warsaw, Poland
[3] Université Paris-Saclay, CEA, CNRS, Institute for Integrative Biology of the Cell (I2BC), 91198, Gif-sur-Yvette, France

Table of contents

Supplementary Figure S1. Cytology of time course experiments

Supplementary Figure S2. RNA-independent interaction between Gtsf1 and Ptiwi09. Gtsf1 localization is unchanged upon *EZL1* KD

Supplementary Figure S3. Gtsf1 is required for efficient DNA elimination and TE silencing.

Supplementary Figure S4. New developing MAC sorting by flow cytometry

Supplementary Figure S5. Estimated size of new developing MAC. Gtsf1 depletion affects H3K9me3 and H3K27me3 enrichment, and Ezl1 levels.

Supplementary Figure S6. Analysis of sRNA populations at different times of autogamy.

Supplementary Figure S7. Non coding maternal transcription is not affected upon *GTSF1* KD.

Supplementary Figure S8. Ema1 is necessary for DNA elimination and scnRNA selection

Supplementary Figure S9. MG132 treatment leads to increased levels of ubiquitinylated proteins and of Ptiwi09, and impairs sexual events and sexual progeny production

Supplementary Table S1. Production of sexual progeny following RNAi-mediated gene silencing

Supplementary Table S2. Ptiwi09 and Gtsf1 interact together and with PRC2

Supplementary Table S3. List of primers used in this study

Supplementary Table S4. Sequencing data and mapping statistics

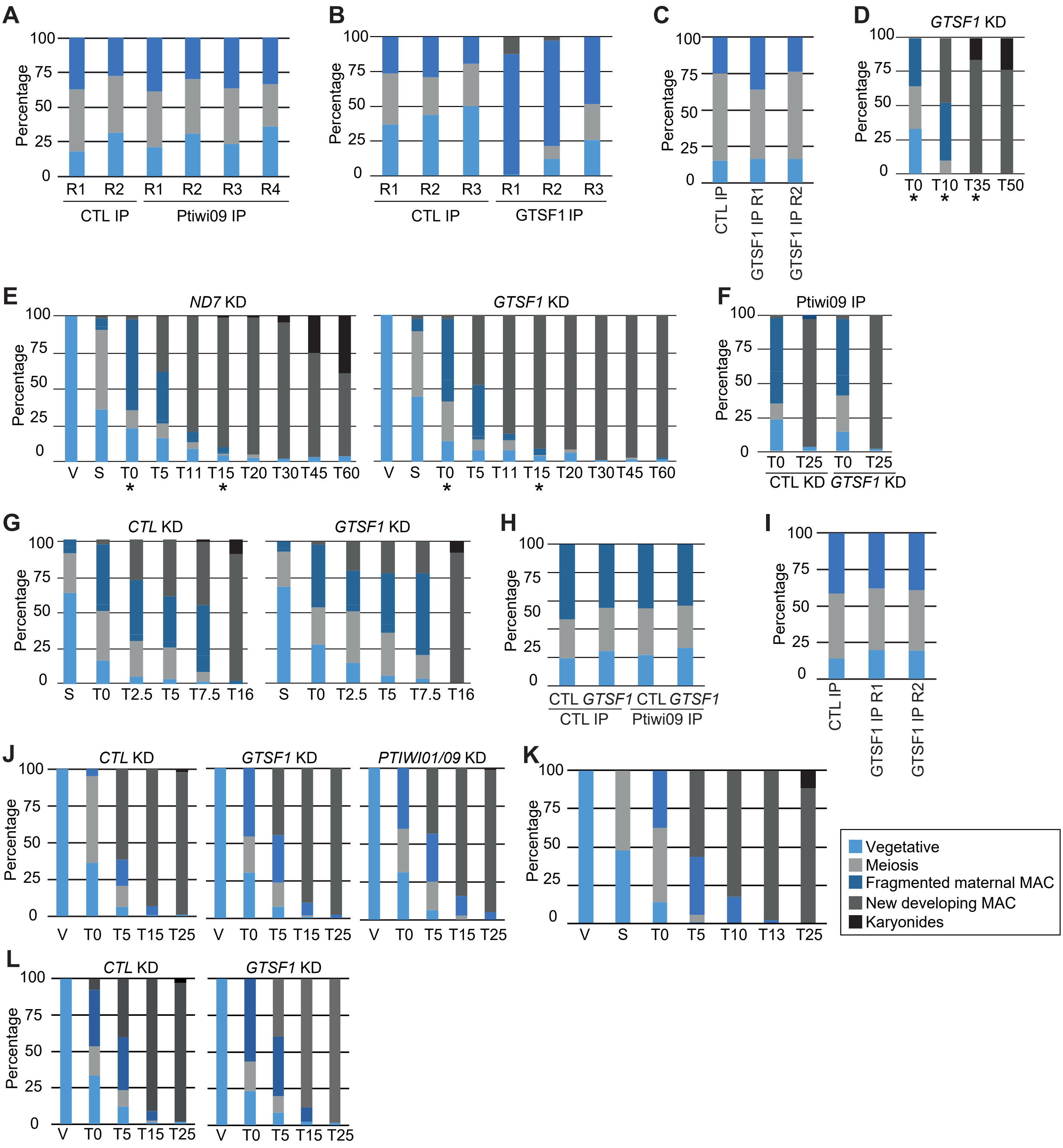

Supplementary Figure S1. Cytology of time course experiments. Progression of autogamy is followed by cytology with DNA staining in the time course experiments. >100 cells were counted in each condition.

A. Cytology of Ptiwi09 IP experiments, Related to Figure 1.
B. Cytology of Gtsf1 IP experiments, Related to Figure 2.
C. Cytology of Gtsf1 IP experiment with RNAse I treatment, Related to Figure 2 and Supplementary Figure S2A.
D. Cytology of time course experiment for sRNA and RNA sequencing in *GTSF1* KD, Related to Figures 4F, 6B-C and Supplementary Figure S6. The star (*) indicates the samples used for sequencing.
E. Cytology of time course experiment to detect scnRNA levels in CTL or *GTSF1* KD, Related to Figure 6A and Supplementary Figure S7. The star (*) indicates the samples used for sequencing.
F. Cytology of time course experiment for sRNA sequencing from Ptiwi09 IP in CTL or *GTSF1* KD, Related to Figure 6D-E.
G. Cytology of time course experiment for Ptiwi09 detection in CTL or *GTSF1* KD, Related to Figure S2C.
H. Cytology of time course experiment for Ptiwi09 IP in CTL or *GTSF1* KD, Related to Figure 9A.
I. Cytology of time course experiment for sRNA sequencing from Gtsf1 IP at T=0 hours after the onset of autogamy, Related to Figure 9B-D and Supplementary Figure S6.
J. Cytology of time course experiment for Ptiwi09 antibody validation, Related to Supplementary Figure S2B.
K. Cytology of time course experiment for Gtsf1 detection by western blot. Related to Figure 3.
L. Cytology of time course experiment for Ptiwi09 detection in CTL or *GTSF1* KD, Related to Figure 7B and S2D.

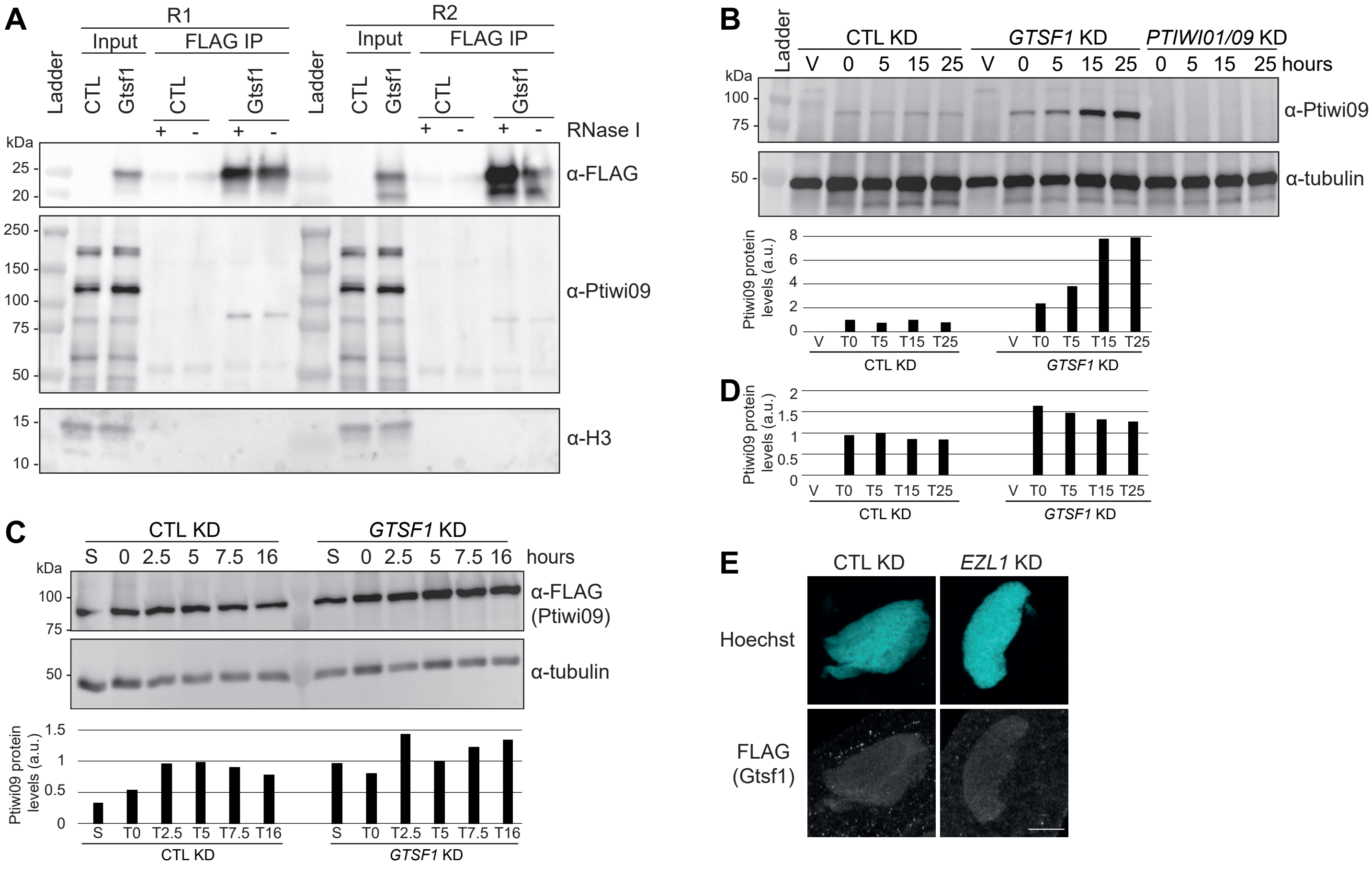

Supplementary Figure S2. RNA-independent interaction between Gtsf1 and Ptiwi09. Gtsf1 localization is unchanged upon *EZL1* KD.

A. Western blot analysis of nuclear extracts of *Paramecium* expressing a 3XFLAG-HA-Gtsf1 functional protein (Gtsf1) or not (CTL) before (input) or after affinity purification (FLAG IP). The affinity purification experiment is performed in presence (+) or absence (-) of an RNase I treatment in two replicates (R1 is shown Figure 2). Anti-FLAG, anti-Ptiwi09 and anti-H3 antibodies are used for detection.

B. Custom Ptiwi09 antibodies validation. Western blot analysis using anti-Ptiwi09 antibodies on *Paramecium* whole cell extracts upon *PTIWI01/09, GTSF1* or *ND7* (CTL) RNAi-mediated silencing (KD) before autogamy (V=vegetative) and at T=0, 5, 15 or 25 hours after the onset of autogamy (Supplementary Figure S1). Anti-tubulin antibodies are used for normalization. Western blot quantification of endogenous Ptiwi09 protein levels is shown below. The level of Ptiwi09 at T=0 hours in the control KD is set up to 1.

C. Western blot analysis of whole cell extracts at different time points (S= starved; T=0; 2.5; 5; 7.5; 16 hours after the onset of autogamy) in *ND7* (CTL) and *GTSF1* KDs with FLAG antibodies to detect 3XFLAG-Ptiwi09 and tubulin antibodies for normalization. Western blot quantification of Ptiwi09 protein levels (FLAG) is shown below. The level of Ptiwi09 at T=7.5 hours in the control KD is set up to 1.

D. Western blot quantification of Ptiwi09 protein levels (FLAG) from Figure 7B. The level of Ptiwi09 at T=5 hours in the control KD is set up to 1.

E. FLAG immunostaining of cells expressing a *3XFLAG-HA-GTSF1* functional transgene at T=0 hours after the onset of autogamy in *ICL7* (CTL) or *GTSF1* KDs. Scale bar, 10 µm.

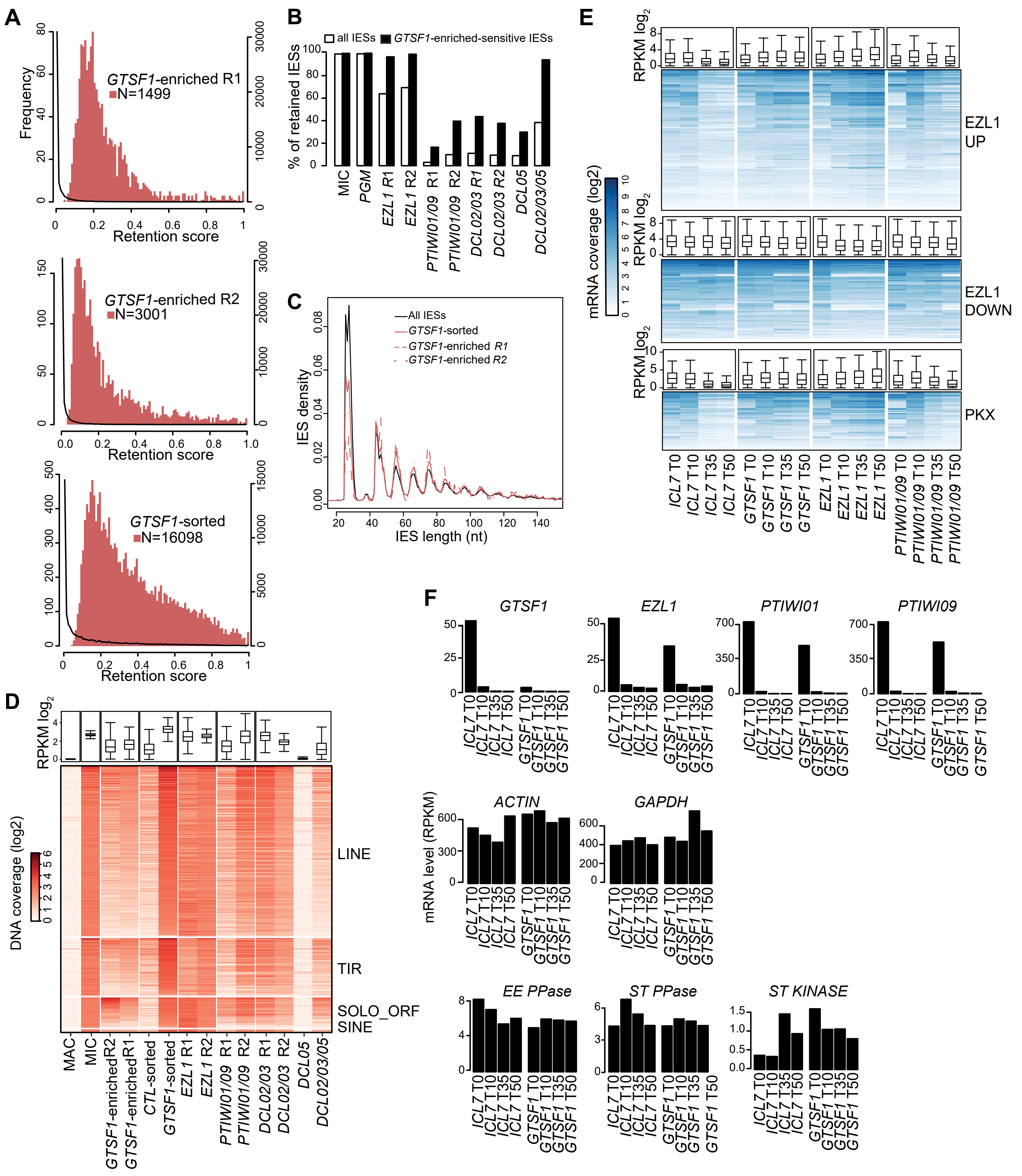

Supplementary Figure S3. Gtsf1 is required for efficient DNA elimination and TE silencing, Related to Figure 4.

A. Histograms of IES retention scores for *GTSF1* KDs. The significantly retained IESs in *GTSF1* KD (2 replicates of *GTSF1*-enriched and 1 replicate of *GTSF1*-sorted) are represented by the red histograms (scale on the left), while the global distribution for all IESs retained in *GTSF1* KD is represented by the black curve (scale on the right).

B. Histogram of the percentage of retained IESs in MIC, *EZL1*, *PTIWI01/09*, *DCL02/03*, *DCL05* and *DCL2/3/5* KDs.

C. IES length distribution for all IESs and IESs retained upon *GTSF1* KD. Note that short IESs are under-represented in *GTSF1* KD.

D. Heatmaps of TE normalized DNA coverage. TE copies are ordered by the mean coverage of *GTSF1*-enriched R1, *GTSF1*-enriched R2 and *GTSF1*-sorted in each family .(LINE n=770, TIR n=261, SOLO ORF n=136 and SINE n=13). The coverage distribution (RPKM log2) for all TE copies is shown as a boxplot. The box shows the first and third quartiles. The median is displayed as a horizontal line. The outliers are not drawn and the whiskers run from the minimum to the maximum value.

E. Heatmaps of gene normalized RNA coverage for up- and down-regulated genes (n=1505 and n=870, respectively) in *EZL1* KD (Frapporti et al., 2019) and up-regulated genes in *PGM-KU80C-XRCC4* KD (PKX, n=628) (Bazin-Gélis et al., 2023). For each dataset, the global mRNA-seq coverage distribution (RPKM log2) is shown as a boxplot. The box shows the first and third quartiles. The median is plotted as a horizontal line. The outliers are not drawn and the whiskers run from the minimum to the maximum value.

F. Barplots of *GTSF1*, *EZL1*, *PTIWI01*, *PTIWI09*, *ACTIN*, *GAPDH*, *EE PPase*, *ST PPase* and *ST KINASE* mRNA levels in *ICL7* (CTL) and *GTSF1* KDs.

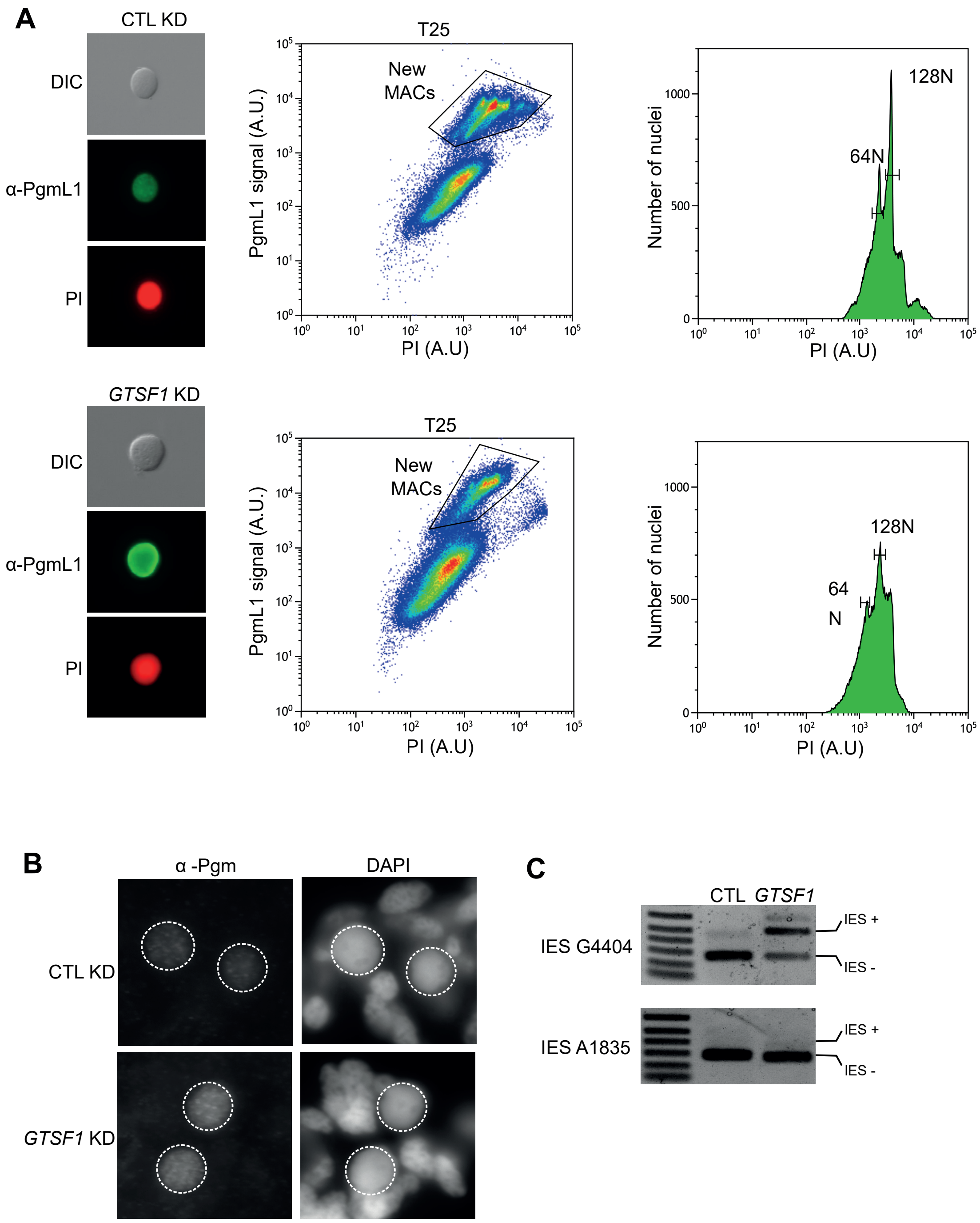

Supplementary Figure S4. New developing MAC sorting by flow cytometry, Related to Figure 4

A. Flow cytometry sorting of α-PgmL1 immunostained nuclei (Zangarelli et al., 2022) from control *ND7* (CTL) and *GTSF1* KD at T=25 hours after the onset of autogamy (DEV3 according to Zangarelli et al., 2022). Left panels: images of the sorted nuclei in phase contrast (DIC), α-PgmL1 labeling and propidium iodide (PI) DNA labeling. Middle panels: plots of PgmL1 fluorescence intensity (y-axis; arbitrary units in log scale) versus PI fluorescence intensity (x-axis). New MACs gating used for nuclei sorting is indicated. Right panels: Histograms of PI-stained nuclei gated in the middle panels. The estimated ploidy level for most prominent peaks is shown.

B. Pgm immunostaining at T=25 hours after the onset of autogamy in control *ND7* (CTL) and *GTSF1* KD. New MACs and fragments of the maternal MAC are stained with DAPI, developing MACs are surrounded by a white dotted circle. The Pgm excision complex localizes in the new MAC upon *GTSF1* KD.

C. PCR around two IES sequences on DNA isolated from sorted new MACs developed upon *ND7* (CTL) and *GTSF1* KD. Excised IES form (IES-) and non-excised form (IES+) are indicated.

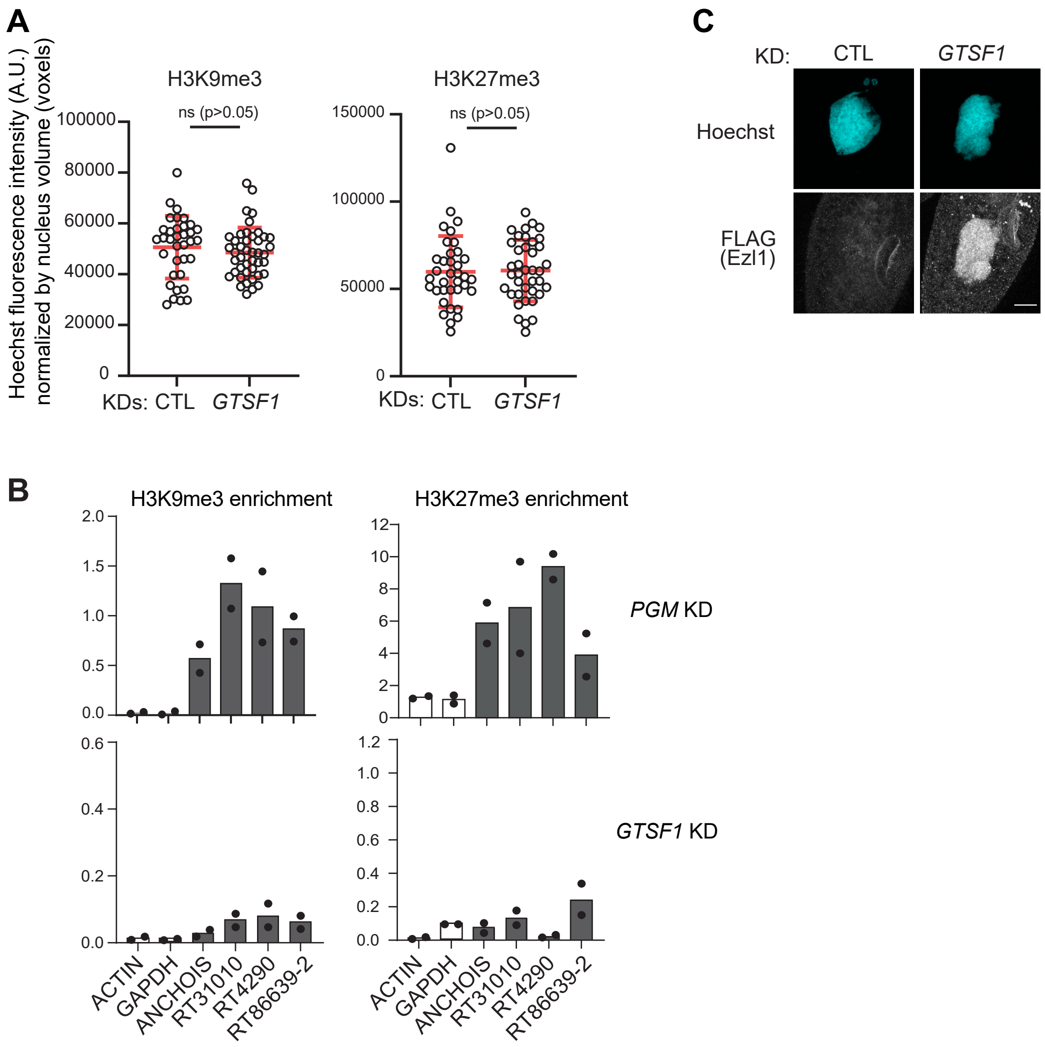

Supplementary Figure S5. Estimated size of new developing MAC. Gtsf1 depletion affects H3K9me3 and H3K27me3 enrichment, and Ezl1 levels. Related to Figure 5

A. Boxplot of estimated nucleus (developing MAC) volume in voxels from the same data as in Figure 5A (T=15 hours). Number of nuclei > 30 in each condition. Estimation of nuclear volume indicated that *CTL* and *GTSF1* KD cell populations were at comparable stages. Bars correspond to mean +/- SD. Mann-Withney statistical test. n.s: non-significant

B. Barplots of H3K9me3 and H3K27me3 enrichment over input (log2) for genes (*ACTIN*, *GAPDH*) (white) and TE copies (grey) determined by ChIP-qPCR upon *PGM* and *GTSF1* KD (2 replicates).

C. FLAG immunostaining of cells expressing a *3XFLAG-HA-EZL1* functional transgene at T=0 hours after the onset of autogamy in *ICL7* (CTL) or *GTSF1* KD. Scale bar, 10 µm.

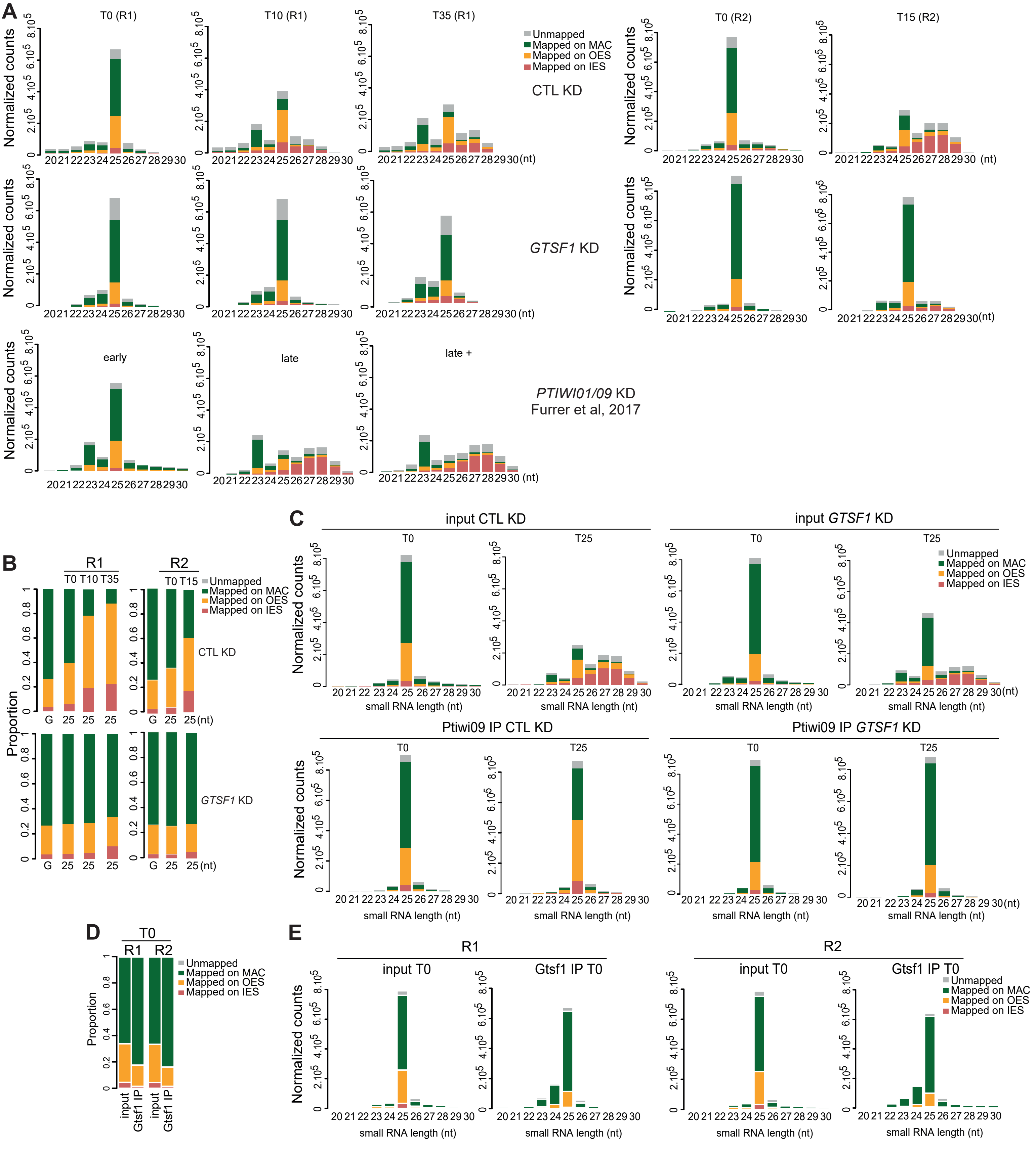

Supplementary Figure S6. Analysis of sRNA populations at different times of autogamy, Related to Figure 6 and Figure 7.

A. Bar plots show the normalized counts for each sample that map the MAC genome, IESs, or OES upon *CTL*, *GTSF1*, or *PTIWI01/09* KD. T=0, 10, 35 hours after the onset of autogamy for replicate 1 (R1, shown in Figure 6C) and T=0 and 15 hours after the onset of autogamy for replicate 2 (R2) are shown.

B. Bar plots show the proportion of 25-nt reads for each sample that map the MAC genome, IESs, or OES upon *CTL* or *GTSF1* KD. T=0, 10, 35 hours after the onset of autogamy for replicate 1 (R1, shown in Figure 6C) and T=0 and 15 hours after the onset of autogamy for replicate 2 (R2) are shown. G: proportion of each category (MAC, OES, IES) in the MIC genome.

C. Bar plots show the normalized counts for each sample before and after Ptiwi09 IP in *ND7* (CTL) or *GTSF1* KD that map the MAC genome, IESs, or OES. T=0 and 25 hours after the onset of autogamy are shown. Related to Figure 6D, E.

D. Bar plots show the proportion of 25-nt reads before (input) and after Gtsf1 IP that map the MAC genome, IESs, or OES. T=0 hours after the onset of autogamy for two replicates (R1 and R2) is shown. R1 is shown in Figure 7E.

E. Bar plots show the normalized counts before (input) and after Gtsf1 IP that map the MAC genome, IESs, or OES. T=0 hours after the onset of autogamy for two replicates (R1 and R2) is shown. R1 is shown in Figure 7F.

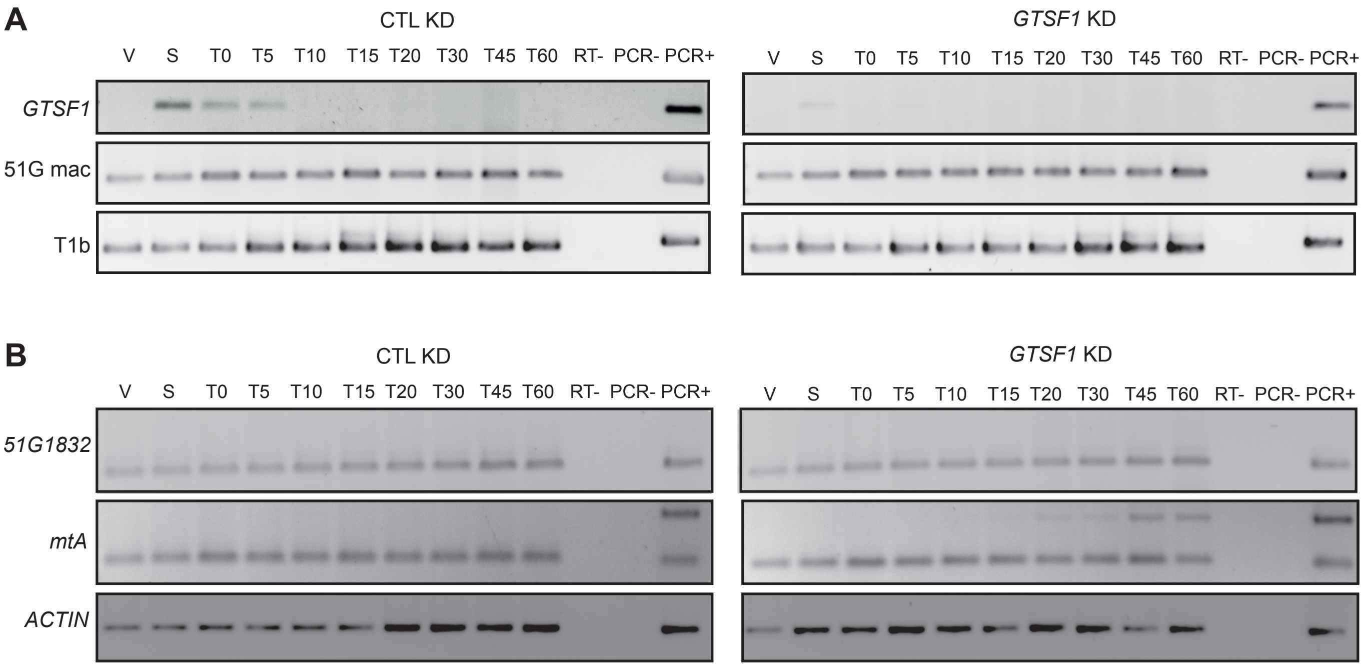

Supplementary Figure S7. Non coding maternal transcription is not affected upon *GTSF1* KD.

Total RNAs, extracted at each time point, were reverse-transcribed and cDNAs were amplified by PCR with gene-specific primers. Loading controls are the constitutively-expressed genes encoding a trichocyst matrix protein (T1b) or actin. Total genomic DNA was used as a positive PCR control. Non-protein-coding RNAs are detected for the G surface antigen gene (51G mac) both during vegetative growth and during sexual events (Lepere et al., 2008). The details of the cytology are displayed in Supplementary Figure S1 panel E.

A. Detection of *GTSF1* and 51G mac ncRNA transcripts by RT-PCR in *ND7* (CTL) and *GTSF1* KD conditions.

B. Detection of *51G1832* and mtA ncRNA transcripts by RT-PCR in *ND7* (CTL) and *GTSF1* KD conditions. Note the presence of an IES+ form for mtA, which is retained in *GTSF1* KD.

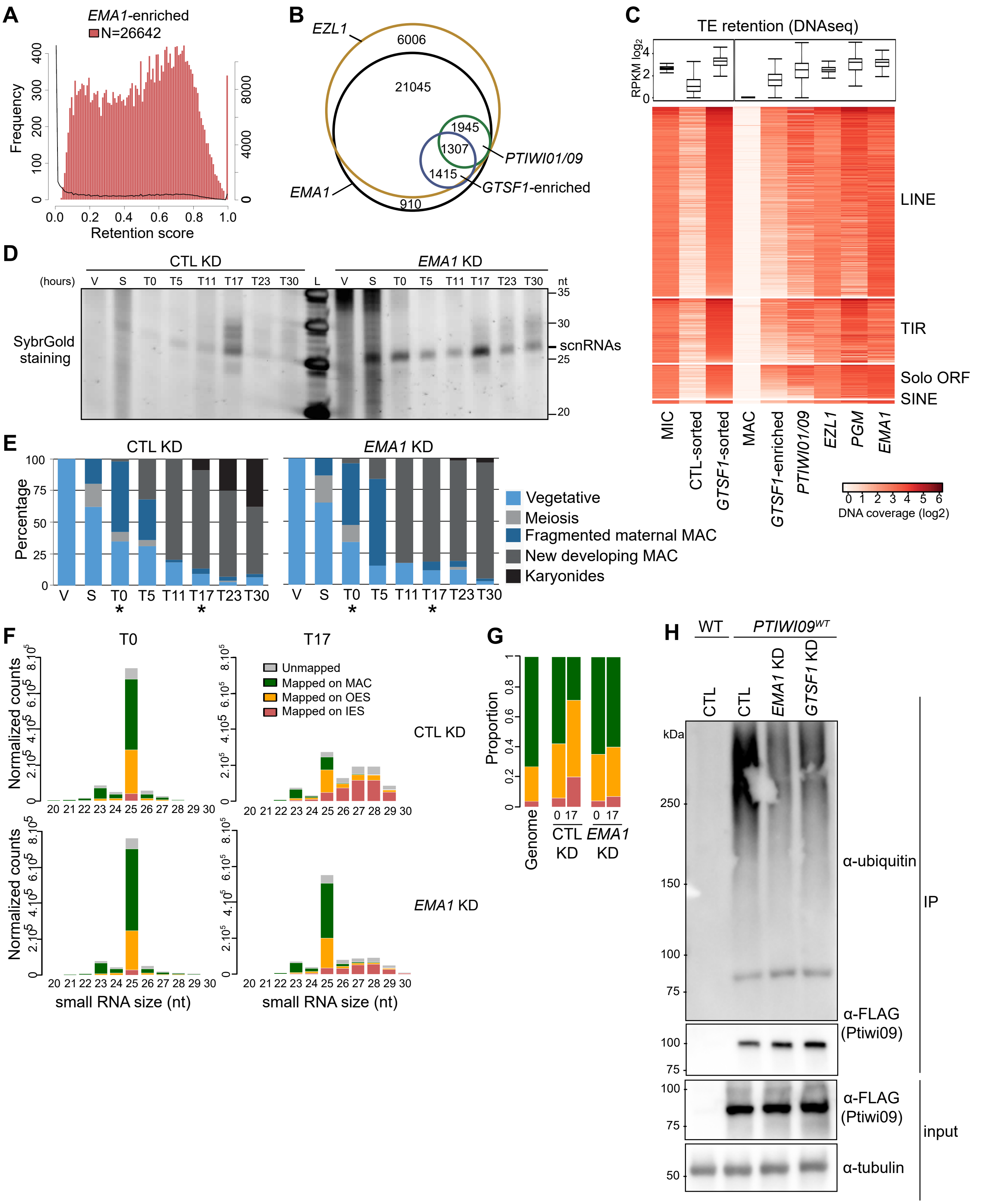

Supplementary Figure S8. Ema1 is necessary for DNA elimination and scnRNA selection.

A. Histograms of IES retention scores upon *EMA1* KD. The significantly retained IESs in *EMA1* KD are represented by the red histograms (scale on the left), while the global distribution for all IESs retained in *EMA1* KD is represented by the black curve (scale on the right).

B. Venn diagram of significantly retained IESs upon different KDs (enriched new MACs in all conditions, 2 *GTSF1*-enriched replicates are combined).

C. Heatmaps of TE normalized DNA coverage. TE copies are ordered by the mean DNA coverage of *GTSF1*-enriched and *GTSF1*-sorted in each family. The boxplots show the coverage (RPKM log_2) for all TE copies.

D. Gel electrophoresis of sRNAs from *ND7* (CTL) and *EMA1* KD cells. Total RNA samples corresponding to different time points (S = starvation, T=0, 5, 11, 17, 23 and 30 hours after the onset of autogamy) were run on a denaturing 15% polyacrylamide-urea gel. After electrophoresis, the gel was stained with SybrGold. L: DNA low molecular weight marker (USB). The 25-nt scnRNAs are indicated.

E. Cytology of the autogamy time course experiment to detect scnRNA levels in CTL or *EMA1* KD (panel D). Progression through autogamy was followed by cytology with DAPI staining. >100 cells were counted in each condition. The star (*) indicates the samples used for sequencing.

F. Analysis of sRNA populations at different time points of autogamy. Bar plots show the normalized counts for each sample that map the MAC genome, IESs, or OES. T=0 and 17 hours after the onset of autogamy.

G. Analysis of 25-nt scnRNA populations in *ND7* (CTL) and *EMA1* KD at different time points (T=0 and 17 hours after the onset of autogamy). Bar plots show the proportion of 25-nt reads for each sample that map the MAC genome, IESs, or MIC-limited sequences. Genome: proportion of each category (MAC, OES, IES) in the MIC genome.

H. Western blot analysis of ubiquitylation of Ptiwi09 immunoprecipitation (IP) at T=0 hours after the onset of autogamy on wildtype cells (WT) and cells expressing *3xFLAG-PTIWI09* transgene ($PTIWI09^{WT}$) in control (empty vector, CTL), upon *EMA1* and *GTSF1* KD. Ptiwi09 detection was performed using FLAG antibodies before (input) and after (IP) Ptiwi09 immunoprecipitation. α-tubulin antibodies were used for normalization.

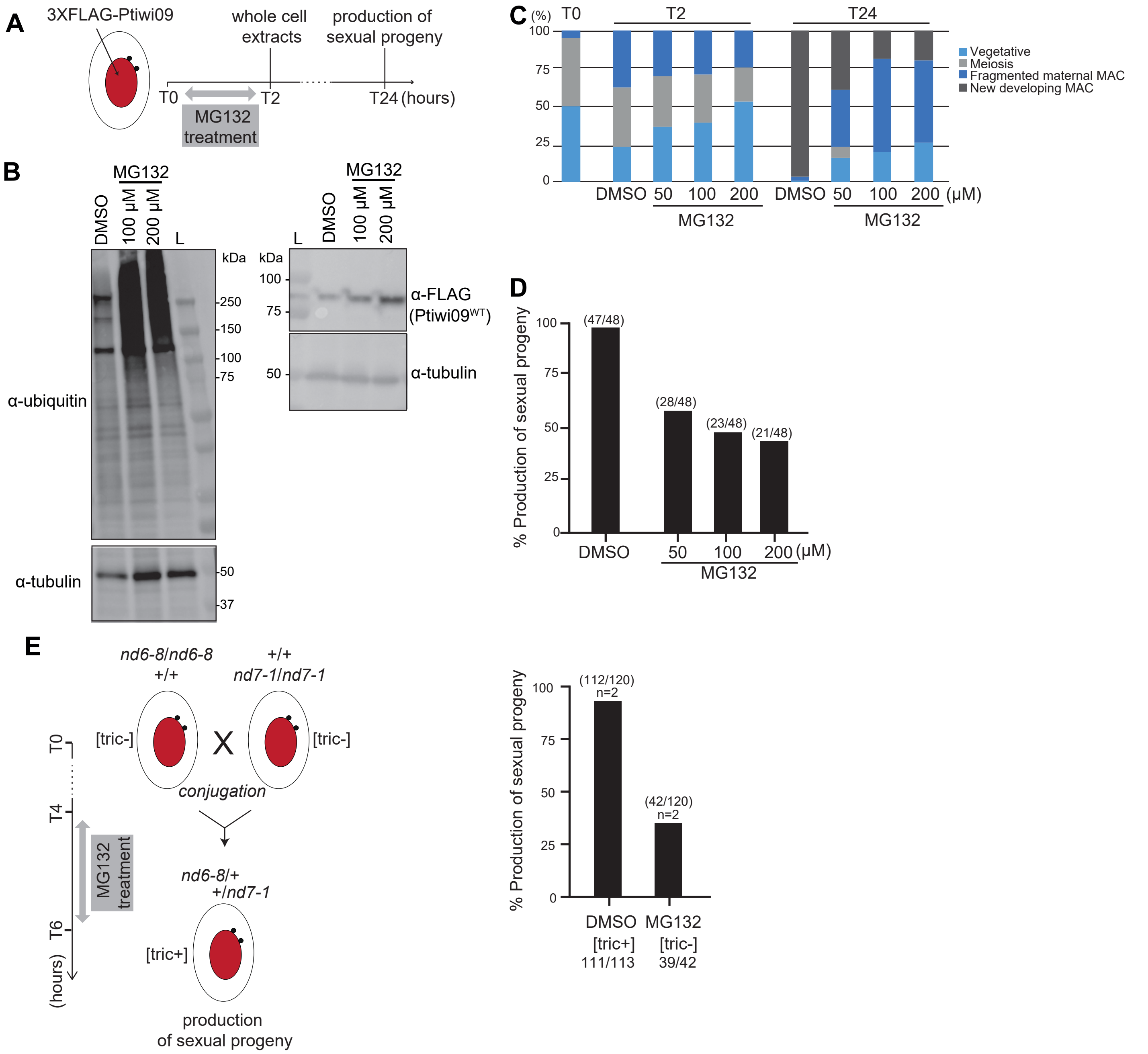

Supplementary Figure S9. MG132 treatment leads to increased levels of ubiquitinylated proteins and of Ptiwi09, and impairs sexual events and sexual progeny production.

A. Experimental design. 3xFLAG-Ptiwi09-transformed cells were grown in standard conditions up to 3000 cells/mL, washed with 10 mM Tris pH 7.4, transferred to exhausted medium (standard medium from which bacteria have been removed) until T=0 hour of autogamy (30% of cells at meiosis), then incubated for 2 hours with DMSO, 50, 100 or 200 µM MG132 (Apollo Scientific). After the 2-hour treatment, cells were washed to remove the drug, and collected to prepare whole cell extracts (panel B), and examine the progression through autogamy by cytology (panel C), while the remaining cells were examined the next day for their cytology (panel C) and for their ability to produce sexual viable progeny (panel D).

B. The levels of ubiquitylated proteins and of Ptiwi09 increase upon MG132 treatment. Western blot analysis was performed on whole cell extracts at T=2 hours with anti-ubiquitin, -FLAG and -tubulin antibodies.

C. Progression of autogamy is altered upon MG132 treatment (T0 to T2). Progression of autogamy is followed by cytology with DNA staining at the indicated time points. Approximately 100 cells were counted in each condition.

D. MG132 treatment impairs the production of viable sexual progeny.

E. Survival and genetic analysis of F1 progeny from a cross between d4.2 mt7 *nd6-8* cells and d4.2 mt8 *nd7-1* cells (Lefort-Tran et al. 1981). 4 hours after mixing, mating pairs were incubated for 2 hours with DMSO or 100 µM MG132, then transferred to exhausted medium without drug to complete conjugation. The histogram shows the number of cells surviving conjugation and the genotype of their MAC, out of 120 cells in each condition. The cell phenotype determined by the MAC is indicated between square brackets below ([tric+]: cells are able to discharge their trichocysts; [tric-]: cells are not able to discharge their trichocysts as the *nd6-8* or *nd7-1* homozygous mutants). The mutant phenotype [tric-] observed in F1 upon MG132 treatment results from a conjugation failure -unsuccessful exchange of gametic nuclei- within a mating pair.

Experiment n°	Transgene	KD gene	sexual progeny	
1 - Production of sexual progeny (Figure 2C)	-	*ICL7*	Alive	30
			Dead	0
		GTSF1#1	Alive	0
			Dead	30
		ICL7	Alive	30
			Dead	0
		GTSF1#1	Alive	0
			Dead	30
2 - Production of sexual progeny (Figure 2C)	-	-	Alive	29
			Dead	1
		GTSF1#1	Alive	0
			Dead	30
3 - Production of sexual progeny (Figure 2C), DNA seq (Figure 4, Supplementary Figure S3), RNA-seq (Figure 4, Supplementary Figure S3, Supplementary Figure S1) and small RNA seq (Figure 6B, Supplementary Figure S6, Supplementary Figure S1) upon *GTSF1* KD	-	*ICL7*	Alive	29
			Dead	1
		GTSF1#1	Alive	0
			Dead	30
4 - Ezl1 localization by immunofluorescence (Supplementary Figure S5)	-	*ICL7*	Alive	13
			Dead	2
		GTSF1#1	Alive	0
			Dead	15
	3XFLAG-HA-EZL1	*ICL7*	Alive	15
			Dead	0
		GTSF1#1	Alive	3
			Dead	12
	3XFLAG-HA-EZL1	*ICL7*	Alive	15
			Dead	0
		GTSF1#1	Alive	2
			Dead	13
5 - Gtsf1 genetic complementation experiments (Figure 2C) and localization at different stages of the sexual cycle (Figure 3)	-	*ICL7*	Alive	30
			Dead	0
		GTSF1#1	Alive	1
			Dead	29
	3XFLAG-HA-GTSF1	*ICL7*	Alive	28
			Dead	2
		GTSF1#1	Alive	29
			Dead	1
	3XFLAG-HA-GTSF1	*ICL7*	Alive	28
			Dead	2
		GTSF1#1	Alive	29
			Dead	1

6 - Gtsf1 IP (Figure 2D, 2E, 2F and 2G)	-	*ICL7*	Alive	30
			Dead	0
		GTSF1#1	Alive	1
			Dead	29
	3XFLAG-HA-GTSF1	*ICL7*	Alive	28
			Dead	2
		GTSF1#1	Alive	29
			Dead	1
	3XFLAG-HA-GTSF1	*ICL7*	Alive	28
			Dead	2
		GTSF1#1	Alive	29
			Dead	1
7 - Production of sexual progeny (Figure 2C)	-	*ICL7*	Alive	29
			Dead	1
		GTSF1#1	Alive	0
			Dead	30
	3XFLAG-HA-GTSF1	*ICL7*	Alive	29
			Dead	1
		GTSF1#1	Alive	14
			Dead	1
	3XFLAG-HA-GTSF1	*ICL7*	Alive	15
			Dead	0
		GTSF1#1	Alive	30
			Dead	0
8 - Ptiwi09 localization (Figure 7A)	*3XFLAG-HA-PTIWI09*	*ICL7*	Alive	20
			Dead	7
		GTSF1#1	Alive	30
			Dead	27
	3XFLAG-HA-PTIWI09	*ICL7*	Alive	30
			Dead	0
		GTSF1#1	Alive	2
			Dead	28
9 - Production of sexual progeny (Figure 2C)	-	*ICL7*	Alive	23
			Dead	7
		GTSF1#1	Alive	0
			Dead	30
10 - Production of sexual progeny (Figure 2C)	-	*ICL7*	Alive	26
			Dead	4
		GTSF1#1	Alive	0
			Dead	30
		ICL7	Alive	28
			Dead	2
		GTSF1#1	Alive	2
			Dead	25

11 - Production of sexual progeny (Figure 2C)	—	*ICL7*	Alive	48
			Dead	0
		GTSF1#1	Alive	0
			Dead	48
		ICL7	Alive	47
			Dead	1
		GTSF1#1	Alive	0
			Dead	48
		ICL7	Alive	46
			Dead	2
		GTSF1#1	Alive	0
			Dead	48
		ICL7	Alive	44
			Dead	4
		GTSF1#1	Alive	2
			Dead	46
12 - Ptiwi09 IP (Figure 1)	3xFLAG-PTIWI09	—	Alive	48
			Dead	0
	3xFLAG-PTIWI09	—	Alive	47
			Dead	1
	3xFLAG-PTIWI09	—	Alive	46
			Dead	2
	3xFLAG-PTIWI09	—	Alive	48
			Dead	0
		—	Alive	48
			Dead	0
		—	Alive	48
			Dead	0
13 - Autogamy time course sRNA (Figure 6A, Supplementary Figure S1) ; sRNA seq (Figure Supplementary Figure S6) ; DNA (Figure 4 and Supplementary Figure S3) ; RT-PCR (Supplementary Figure S7) upon GTSF1 KD	—	*ND7*	Alive	47
			Dead	1
		GTSF1#2	Alive	3
			Dead	45
14 -GTSF1-sorted DNA seq (Figure 4, Figure Supplementary Figure S3 and Supplementary Figure S3)	—	*ND7*	Alive	47
			Dead	1
		GTSF1#2	Alive	2
			Dead	46
15 - Ptiwi09 RNA IP (Figure 6, Figure Supplementary Figure S6)	3xFLAG-PTIWI09	*ICL7*	Alive	48
			Dead	0
		GTSF1#2	Alive	2
			Dead	46
	—	*ICL7*	Alive	48
			Dead	0
		GTSF1#2	Alive	0
			Dead	48

16 - Autogamy time course (Figure 7B)	3xFLAG-PTIWI09	*L4440*	Alive	48
			Dead	0
		GTSF1#2	Alive	0
			Dead	48
	—	*L4440*	Alive	45
			Dead	3
		GTSF1#2	Alive	0
			Dead	48
17 - Ptiwi09 IP (Figure 9A)	3xFLAG-PTIWI09	*ICL7*	Alive	48
			Dead	0
		GTSF1#2	Alive	1
			Dead	47
	—	*ICL7*	Alive	48
			Dead	0
		GTSF1#2	Alive	2
			Dead	46
18 - PGM staining (Supplementary Figure S4)	—	*ND7*	Alive	48
			Dead	0
		GTSF1#2	Alive	1
			Dead	47
19 - DNA seq upon Ptiwi01/09 KD (R1) (Supplementary Figure S3)	—	*PTIWI01/09*	Alive	
			Dead	63%
20 - Gtsf1 RNA IP (Figure 9B-D)	*3XFLAG-HA-GTSF1*	—	Alive	48
			Dead	0
	3XFLAG-HA-GTSF1	—	Alive	48
			Dead	0
	—	—	Alive	48
			Dead	0
21 - Gtsf1 IP (Figure 2 and Supplementary Figure S2)	*3XFLAG-HA-GTSF1*	—	Alive	48
			Dead	0
	3XFLAG-HA-GTSF1	—	Alive	48
			Dead	0
	—	—	Alive	48
			Dead	0
22 - Production of sexual progeny (Supplementary Figure S2B)	—	*ND7*	Alive	46
			Dead	2
		GTSF1#2	Alive	0
			Dead	48
		PTIWI01/09	Alive	1
			Dead	47

23 - Gtsf1 localization by immunofluorescence (Supplementary Figure S2)	*3XFLAG-HA-GTSF1*	*ICL7*	Alive	28
			Dead	2
		EZL1	Alive	0
			Dead	30
	3XFLAG-HA-GTSF1	*ICL7*	Alive	30
			Dead	0
		EZL1	Alive	0
			Dead	30
	—	*ICL7*	Alive	27
			Dead	3
		EZL1	Alive	0
			Dead	30
24- Production of sexual progeny (Figure 8)	*3xFLAG-HA-GTSF1*	*ICL7*	Alive	48
			Dead	0
		PTIWI01/09	Alive	2
			Dead	46
		EMA1	Alive	0
			Dead	48
	3xFLAG-HA-GTSF1	*ICL7*	Alive	47
			Dead	1
		PTIWI01/09	Alive	3
			Dead	45
		EMA1	Alive	0
			Dead	48
	—	*ICL7*	Alive	48
			Dead	0
		PTIWI01/09	Alive	2
			Dead	46
		EMA1	Alive	0
			Dead	48
25- Production of sexual progeny (Figure 8)	*3xFLAG-PTIWI09*	*ICL7*	Alive	48
			Dead	0
		GTSF1#2	Alive	0
			Dead	48
		EMA1	Alive	0
			Dead	48
	3xFLAG-PTIWI09	*ICL7*	Alive	48
			Dead	0
		GTSF1#2	Alive	0
			Dead	48
		EMA1	Alive	0
			Dead	48
	—	*ICL7*	Alive	48
			Dead	0
		GTSF1#2	Alive	0
			Dead	48
		EMA1	Alive	0
			Dead	48

26 - survival after MG132 treatment during autogamy (Supplementary Figure S9)	*3xFLAG-PTIWI09*	DMSO	Alive	47
			Dead	1
		MG132 (50 µM)	Alive	28
			Dead	20
		MG132 (100 µM)	Alive	23
			Dead	25
		MG132 (200 µM)	Alive	21
			Dead	27
27 - survival after MG132 treatment during conjugation (Supplementary Figure S9)	*d4.2 ND6-8 mt7 X d4.2 ND7-1 mt8 (3xFLAG-Ptiwi09)*	DMSO	Alive	59
			Dead	1
		MG132 (100 µM)	Alive	30
			Dead	30
	d4.2 ND6-8 mt7 X d4.2 ND7-1 mt8 (3xFLAG-Ptiwi09)	DMSO	Alive	53
			Dead	7
		MG132 (100 µM)	Alive	12
			Dead	48
28 - Gtsf1 detection by western blot (Figure 3B)	NI	—	Alive	46
			Dead	2
	3XFLAG-HA-GTSF1	—	Alive	47
			Dead	1
29 - Autogamy time course, sRNA-seq and sRNA seq upon *EMA1* KD (Supplementary Figure S8)	—	*ND7*	Alive	47
			Dead	1
		EMA1	Alive	2
			Dead	46
30 - DNA seq upon *EMA1* KD (Supplementary Figure S8)	—	*EMA1*	Alive	0
			Dead	48
31 - Production of sexual progeny (Figure 8)	*3xFLAG-HA-GTSF1*	*L4440*	Alive	48
			Dead	0
		PTIWI01/09	Alive	1
			Dead	47
		EMA1	Alive	0
			Dead	48
	—	*L4440*	Alive	48
			Dead	0
		PTIWI01/09	Alive	2
			Dead	46
		EMA1	Alive	0
			Dead	48
32 - Production of sexual progeny (Figure 8)	*3xFLAG-Ptiwi09*	*L4440*	Alive	48
			Dead	0
		GTSF1#2	Alive	3
			Dead	45
		EMA1	Alive	2
			Dead	46
	—	*L4440*	Alive	47
			Dead	1
		GTSF1#2	Alive	1
			Dead	47

		EMA1	Alive	0
			Dead	48
33 - Production of sexual progeny (Figure 7, Supplementary Figure S2)	*3xFLAG-Ptiwi09*	*L4440*	Alive	48
			Dead	0
		GTSF1#2	Alive	0
			Dead	48
34 - Production of sexual progeny (Ptiwi09 ubiquitinylation upon *EMA1* KD and *GTSF1* KD) (Supplementary Figure S8)	*3xFLAG-Ptiwi09*	*L4440*	Alive	48
			Dead	0
		EMA1	Alive	1
			Dead	47
		GTSF1#2	Alive	0
			Dead	48
	–	*L4440*	Alive	48
			Dead	0

Supplementary Table S1. Production of sexual progeny following RNAi-mediated gene silencing.

In each experiment, the number of cells that survived or died is indicated.

	Name	Reference	MW (kDa)	Unique peptide	Log^2 FC	-Log10 p-value
Ptiwi09 IP	Caf1	PTET.51.1.P0780031	45.62	8	13.06	1.85
	Eap1	PTET.51.1.P1310069	20.10	3	5.42	3.14
	Eed	PTET.51.1.P0240079	43.92	3	16.19	4.81
	Ezl1	PTET.51.1.P1740049	69.69	6	11.38	1.36
	Gtsf1	PTET.51.1.P0490019	18.67	2	3.91	2.97
	Pdsg1	PTET.51.1.P0300085	37.98	3	9.56	1.32
	P0390035	PTET.51.1.P0390035	48.21	2	13.60	3.93
	Ptiwi03	PTET.51.1.P0030302	89.92	2	17.44	2.46
	Ptiwi09 (bait)	PTET.51.1.P0660118	87.65	11	3.92	1.36
	Rf2	PTET.51.1.P1190062	74.74	9	13.02	1.72
	Rf4	PTET.51.1.P0570234	62.62	13	7.62	3.87
	Rpb2	PTET.51.1.P0480005	139.59	3	13.07	3.83
Gtsf1 IP	Caf1	PTET.51.1.P0780031	45.62	25	5.04	2.37
	Eap1	PTET.51.1.P1310069	20.10	5	5.52	2.02
	Eed	PTET.51.1.P0240079	43.92	18	4.48	2.11
	Ema1b	PTET.51.1.P0080253	174.48	30	3.68	1.49
	Ezl1	PTET.51.1.P1740049	69.69	30	4.88	2.08
	Gtsf1 (bait)	PTET.51.1.P0490019	18.67	11	9.48	1.55
	P0850169	PTET.51.1.P0850169	57.84	2	6.94	4.78
	P1010056	PTET.51.1.P1010056	40.59	2	5.79	1.49
	Pdsg1	PTET.51.1.P0300085	37.98	23	6.35	2.88
	Ptiwi01	PTET.51.1.P0710112	87.72	8	7.57	1.34
	Ptiwi03	PTET.51.1.P0030302	89.92	12	7.71	2.49
	Ptiwi09	PTET.51.1.P0660118	87.65	10	8.68	1.98
	Rf2	PTET.51.1.P1190062	74.74	48	5.66	1.92
	Rf4	PTET.51.1.P0570234	62.62	40	5.27	1.99
	Rpb2	PTET.51.1.P0480005	139.59	18	2.06	1.55
	Rpb3a	PTET.51.1.P0510184	35.29	18	2.27	1.50
	Rpb3b	GSPATG00038663001	33.99	10	2.35	1.34
	Suz12.like	PTET.51.1.P0190277	34.65	17	4.48	2.07

Supplementary Table S2. Ptiwi09 and Gtsf1 interact together and with PRC2.

Related to Figures 1 and 2. Top differential proteins in Flag IP compared to control IP for Ptiwi09 and for Gtsf1 IPs. A p-value inferior than 0.05 and a fold change superior than 2 were used to filter differential significant candidates. -Log10(p-value) > 1.3 is required to get a p-value < 0.05. Only the protein with the same expression profile than Ptiwi09 and Gtsf1 (early peak) were selected.

Name	Sequence (5' to 3')	Locus (ID)	Source	Application
Actin_qPCR_for	TGAAGCTCCAATGAATCCAA	Actin 1-1 (PTET.51.1.G0130204)	Frapporti et al., 2019	ChIP-qPCR
Actin_qPCR-rev	TCCTGAAGCATAGAGTGAGA	Actin 1-1 (PTET.51.1.G0130204)	Frapporti et al., 2019	ChIP-qPCR
GAPDH_qPCR_F2	ATTTTGGTATTGTTGAGGGT	GAPDH (PTET.51.1.G0380195)	Frapporti et al., 2019	ChIP-qPCR
GAPDH_qPCR_R2	CTCCAGTCTTTTCCACCTTT	GAPDH (PTET.51.1.G0380195)	Frapporti et al., 2019	ChIP-qPCR
Anchois.173_F2	TTCCAAGCTGATTCGATTATTAAA	Anchois B (IESPGM.PTET51.1.173.70900)	Frapporti et al., 2019	ChIP-qPCR
Anchois.173_R2	ACTTCTTGTTTCATTTGTTAGACT	Anchois B (IESPGM.PTET51.1.173.70900)	Frapporti et al., 2019	ChIP-qPCR
Oligo #551 (RT31010)	ACAAGATTGACCAGGACTTATT	RT31010 (ms4410_NODE_3768_length_13900 _cov_21.582806_RT31010_Group4_nonLTR:ClassI:LINE)	Frapporti et al., 2019	ChIP-qPCR
Oligo #552 (RT31010)	ATATCATTCACTCCTGCAATCT	RT31010 (ms4410_NODE_3768_length_13900 _cov_21.582806_RT31010_Group4_nonLTR:ClassI:LINE)	Frapporti et al., 2019	ChIP-qPCR
Oligo #559 (RT42890)	TTAATTGAAGGCGAAGAAAGAC	RT42890 (ms1831_NODE_10132_length_49470 _cov_21.140064_RT42890_Group4_nonLTR:ClassI:LINE)	Frapporti et al., 2019	ChIP-qPCR
Oligo #560 (RT42890)	TTAATTGAAGGCGAAGAAAGAC	RT42890 (ms1831_NODE_10132_length_49470 _cov_21.140064_RT42890_Group4_nonLTR:ClassI:LINE)	Frapporti et al., 2019	ChIP-qPCR
Oligo #723 (RT48639-2)	ATCATCTTTCCCTCACATCG	RT48639-2 (ms4963_NODE_3562_length_10237 _cov_31.527792_RT48639exp_Group2_nonLTR:ClassI:LINE)	Frapporti et al., 2019	ChIP-qPCR
Oligo #724 (RT48639-2)	AGATTTCACGCTTCAGTTCT	RT48639-2 (ms4963_NODE_3562_length_10237 _cov_31.527792_RT48639exp_Group2_nonLTR:ClassI:LINE)	Frapporti et al., 2019	ChIP-qPCR
Oligo #761 (RT48639-1)	GACTATGCTGACGATCTTGT	(ms6074_NODE_8863_length_4403 _cov_18.113333_RT48639old_Group2_non-LTR:ClassI:LINE)	Frapporti et al., 2019	ChIP-qPCR
Oligo #762 (RT48639-1)	TTCTGATTGCCATAACACCA	(ms6074_NODE_8863_length_4403 _cov_18.113333_RT48639old_Group2_non-LTR:ClassI:LINE)	Frapporti et al., 2019	ChIP-qPCR
Oligo #751 (EE PPase)	CTTAGTGGGGTAGAATGAGCA	PTET.51.1.G1020193	Frapporti et al., 2019	ChIP-qPCR
Oligo #752 (EE PPase)	GACTTCTGCTTTCTTTTCTGCA	PTET.51.1.G1020193	Frapporti et al., 2019	ChIP-qPCR
Oligo #753 (ST PPase)	GGAGAGGGAAAGATAAGAGT	PTET.51.1.G1240023	Frapporti et al., 2019	ChIP-qPCR
Oligo #754 (ST PPase)	CCACTCCTTGAATTTGAGGA	PTET.51.1.G1240023	Frapporti et al., 2019	ChIP-qPCR
Oligo #759 (ST Kinase)	GAAGTAGGTATTATCGTGCC	PTET.51.1.G1270115	Frapporti et al., 2019	ChIP-qPCR
Oligo #759 (ST Kinase)	ACCATGTAAACAATTCAAGCA	PTET.51.1.G1270115	Frapporti et al., 2019	ChIP-qPCR
Oligo #769 (Helicase)	AGAGAGAGACTTCGTGATGA	PTET.51.1.G1070046	Frapporti et al., 2019	ChIP-qPCR
Oligo #770 (Helicase)	CAACTTGGGCATGTCAAAAT	PTET.51.1.G1070046	Frapporti et al., 2019	ChIP-qPCR
Oligo#1341 (42AB1)	TGGAGTTTGGTGCAGAAGC	42AB region 1 (chr2R: 6449409–6449518)	Casier et al., 2019	ChIP-qPCR
Oligo#1342 (42AB1)	AGCCGTGCTTTATGCTTTACT	42AB region 1 (chr2R: 6449409–6449518)	Casier et al., 2019	ChIP-qPCR

Oligo#1339 (RPL32)	CCGCTTCAAGGGACAGTATCTG	RPL32 FLYB: FBgn0002626	Casier et al., 2019	ChIP-qPCR
Oligo#1340 (RPL32)	ATCTCGCCGCAGTAAACGC	RPL32 FLYB: FBgn0002626	Casier et al., 2019	ChIP-qPCR
expGTSF1U	ATGTAATTAAAATTGAAATCCTACGAACTAG	GTSF1 (PTET.51.1.G0490019)	this study	RT-PCR
expGTSF1L	GTTTAATTCTTTTGACCGAGGACTC	GTSF1 (PTET.51.1.G0490019)	this study	RT-PCR
T1b-3'	TTGAGTTGGGATTTGACATAATCGGTGAA	T1b (PTET.51.1.G0980135)	Maliszewska-Olejniczak et al., 2015	RT-PCR
T1b-5' (2)	TCTAATTAAACCAAGAACACGCTGAATTCC	T1b (PTET.51.1.G0980135)	Maliszewska-Olejniczak et al., 2015	RT-PCR
51G18	ACTGTTGCTACACATTGTGCATATGTTACT	51G mac	Maliszewska-Olejniczak et al., 2015	RT-PCR
51G17	GATCAAGTCCAGTTCCTGTTATAGAACTAC	51G mac	Maliszewska-Olejniczak et al., 2015	RT-PCR
G1832	GCTATAACTCTTGAAGCTGCTTGTAATATG	51G	Lhuillier-Akakpo et al., 2014	RT-PCR
G1832	TTGTCAATGAGCCATTAACAGTTGCTGGAT	51G	Lhuillier-Akakpo et al., 2014	RT-PCR
ActinF	AGACCACCCAGCTCTTTTGA	Actin 1-1 (PTET.51.1.G0130204)	this study	RT-PCR
ActinR	TTGGGACTGTGTGTGAGACA	Actin 1-1 (PTET.51.1.G0130204)	this study	RT-PCR
mtApromF	CTTATTCTGCCTTCTCTTGAAATGC	mtA	this study	RT-PCR
mtApromR	AGGTCATCTCTTTCATTAAATTCCT	mtA	this study	RT-PCR

Supplementary Table S3. List of primers used in this study

Sequencing	Sample	Label	ENA Accession	Reference	Number of reads	Aligned reads on the MAC		Aligned reads on the MIC	
DNAseq	KLEB	MAC	ERS452529	Lhuillier-Akakpo et al. 2014	106 056 122	104 443 929	98%	105 570 391	100%
DNAseq	PTET_ND7_RNAi_T25_AlgFACS_JkN_REGN50	ND7_Alg	ERS16327826	this study	38 321 642	35 879 076	94%	38 065 563	99%
DNAseq	PTET_GTSF1L-RNAi_TotalDNA_d4_JKN	GTSF1L	ERS16327827	this study	121 834 466	116 955 124	96%	121 634 908	100%
DNAseq	PTET_ZF1_RNAi_gDNA_T50_DUHA-166	ZF1	ERS16327828	this study	58 868 356	55 622 433	94%	58 332 259	99%
DNAseq	PTET_GTSF1L_RNAi_T25_AlgFACS_JkN_REGN51	GTSF1L_Alg	ERS16327829	this study	58 418 092	47 635 163	82%	57 949 376	99%
DNAseq	PTET_EMA1_RNAi_HEL_S9	EMA1	ERS21121357	this study	111 229 478	88 982 195	80%	108 461 199	98%
DNAseq	MicGSC_BCP_AAIOSF_2_HiSeq	MIC	ERX4616645	Sellis et al. 2021	181 407 606	153 120 470	84%	179 098 715	99%
DNAseq	PGM-1_FACS_ANLG	PGM	SAMN05323661	Guérin et al, 2017	115 558 914	101 303 406	88%	115 259 143	100%
DNAseq	Ezl174-1_RNAi_r1_r2	EZL_r1	ERX466733	Lhuillier-Akakpo et al. 2014	99 695 690	87 432 783	88%	97 164 770	97%
DNAseq	Ezl174-2_RNAi_r1	EZL_r2	ERS452532	Lhuillier-Akakpo et al. 2014	92 725 940	82 152 917	89%	92 446 907	100%
DNAseq	PTIWI19_RNAi_r1	PTIWI19_r1	ERS16327830	this study	49 410 804	46 824 351	95%	48 884 765	99%
DNAseq	PTET_PTIWI_1_9_KD_DNA_ERR1918503	PTIWI19_r2	ERS1656548	Furrer et al. 2017	118 338 520	108 845 895	92%	117 994 275	100%
DNAseq	DCL2_3_RNAi_r1_HBJ-1	DCL2_3_r1	PRJNA184719	Sandoval et al. 2014	100 445 166	89 354 921	89%	99 646 333	99%
DNAseq	Dcl2-3_RNAi_r2	DCL2_3_r2	ERX466736	Lhuillier-Akakpo et al. 2014	99 744 888	92 915 718	93%	99 600 735	100%
DNAseq	DCL5_RNAi_r1_HBJ-2	DCL5	PRJNA184719	Sandoval et al. 2014	90 105 744	88 212 339	98%	89 877 717	100%
DNAseq	DCL235_KD_MaN	DCL235	SAMEA3726521	Swart et al. 2017	54 294 800	51 349 502	95%	53 581 515	99%
mRNAseq	ICL7_T0_RNA_DUHA140	ICL7_T0	ERS6679030	Miro-Pina et al. 2022	79765916	78958586	99%	77571546	97%
mRNAseq	ICL7_T10_RNA_DUHA141	ICL7_T10	ERS6679031	Miro-Pina et al. 2022	86517736	85652820	99%	84056363	97%
mRNAseq	ICL7_T35_RNA_DUHA142	ICL7_T35	ERS6679032	Miro-Pina et al. 2022	109355820	108387011	99%	106333998	97%
mRNAseq	ICL7_T50_RNA_DUHA143	ICL7_T50	ERS6679033	Miro-Pina et al. 2022	100914984	99997163	99%	98007556	97%
mRNAseq	PGM-T2_mRNA_CACTCA	PGM_T2	ERS14842492	Bazin-Gélis et al. 2023	81069812	77132399	95%	74844554	92%
mRNAseq	PGM-T10_mRNA_CTCAGA	PGM_T10	ERS14842490	Bazin-Gélis et al. 2023	71443788	70294491	98%	68207123	95%
mRNAseq	PGM-T30_mRNA_ATTCCT	PGM_T30	ERS14842493	Bazin-Gélis et al. 2023	80832994	75672689	94%	73986877	92%
mRNAseq	PGM-T40_mRNA_CACGAT	PGM_T40	ERS14842494	Bazin-Gélis et al. 2023	79637140	77396584	97%	76217304	96%
mRNAseq	PTET_RNAs_ZF1-T0_DUHA161	ZF1_T0	ERS16327831	this study	123476646	121939263	99%	119767868	97%
mRNAseq	PTET_RNAs_ZF1-T10_DUHA162	ZF1_T10	ERS16327832	this study	118472868	116661852	98%	114875670	97%
mRNAseq	PTET_RNAs_ZF1-T35_DUHA163	ZF1_T35	ERS16327833	this study	122393108	120117010	98%	119019613	97%
mRNAseq	PTET_RNAs_ZF1-T50_DUHA164	ZF1_T50	ERS16327834	this study	124817826	122582514	98%	121512408	97%
mRNAseq	EZL1_T0_RNA_DUHA144	EZL1_T0	ERS6679026	Miro-Pina et al. 2022	72271092	71578739	99%	70385457	97%
mRNAseq	EZL1_T10_RNA_DUHA145	EZL1_T10	ERS6679027	Miro-Pina et al. 2022	69918912	68772590	98%	68009496	97%
mRNAseq	EZL1_T35_RNA_DUHA146	EZL1_T35	ERS6679028	Miro-Pina et al. 2022	107514064	99201883	92%	104563941	97%
mRNAseq	EZL1_T50_RNA_DUHA147	EZL1_T50	ERS6679029	Miro-Pina et al. 2022	95433848	86764000	91%	92662006	97%
mRNAseq	ARN_PTIWI1-9_71a-T0_S4 (DUHA90)	PTIWI1_9_T0	ERS6678315	Miro-Pina et al. 2022	105639316	104163879	99%	102551416	97%
mRNAseq	ARN_PTIWI1-9_71a-T10_S5 (DUHA91)	PTIWI1_9_T10	ERS6678316	Miro-Pina et al. 2022	85631710	84289504	98%	83276546	97%
mRNAseq	ARN_PTIWI1-9_71a-T35_S6 (DUHA92)	PTIWI1_9_T35	ERS6678317	Miro-Pina et al. 2022	97923744	95405812	97%	95157309	97%

mRNAseq	ARN_PTIWI1-9_71a-T50_S7 (DUHA93)	PTIWI1_9_T50	ERS6678318	Miro-Pina et al. 2022	97927868	93022880	95%	95185568	97%
sRNAseq	ICL7T0_ATCACG_L006_007_R1_001	ICL7 T0	PRJEB46608	Miro-Pina et al. 2022	14640580	7232735	49%	11 496 959	79%
sRNAseq	ICL7T10_CGATGT_L006_007_R1_001	ICL7 T10	ERS14549878	Miro-Pina et al. 2023	21656218	4800906	22%	14 886 100	69%
sRNAseq	ICL7T35_TGACCA_L006_007_R1_001	ICL7 T35	ERS14549879	Miro-Pina et al. 2023	5810542	1178480	20%	3 969 579	68%
sRNAseq	sRNA_Control_GTSF1L_T0_JKN	CTL T0	ERS16327835	this study	7470729	3977980	53%	6 349 560	85%
sRNAseq	sRNA_Control_GTSF1L_T15_JKN	CTL T15	ERS16327836	this study	6506248	1114163	17%	5 162 530	79%
sRNAseq	sRNA_GTSF1L_T0_JKN	GTSF1L T0	ERS16327837	this study	6941745	4685083	67%	6 259 542	90%
sRNAseq	sRNA_GTSF1L_T15_JKN	GTSF1L T15	ERS16327838	this study	5915826	3488112	59%	5 215 627	88%
sRNAseq	sRNAs_ZF1_T0_DUHA184	ZF1 T0	ERS16327839	this study	22759952	11888719	52%	16 188 772	71%
sRNAseq	sRNAs_ZF1_T10_DUHA185	ZF1 T10	ERS16327840	this study	26350856	13329934	51%	18 727 289	71%
sRNAseq	sRNAs_ZF1_T35_DUHA186	ZF1 T35	ERS16327841	this study	18305725	7956240	43%	12 645 019	69%
sRNAseq	PTET_EMA1_DO_ND7_T0_S55_R1_001	ND7 T0	ERS21121360	this study	2938060	1457419	50%	2 347 107	80%
sRNAseq	PTET_EMA1_DO_ND7_T17_S56_R1_001	ND7 T17	ERS21121361	this study	4052284	596301	15%	3 200 184	79%
sRNAseq	PTET_EMA1_DO_220_T0_S57_R1_001	EMA1 T0	ERS21121358	this study	3118371	1793933	58%	2 648 363	85%
sRNAseq	PTET_EMA1_DO_220_T17_S58_R1_001	EMA1 T17	ERS21121359	this study	3812312	1527798	40%	3 141 034	82%
sRNAseq	sRNA_ICL-RNAi_Input_T0_JkN_S149	Input T0 CTL	ERS16327842	this study	9081430	5639683	62%	8 216 558	90%
sRNAseq	sRNA_ICL-RNAi_Input_T25_JkN_S150	Input T25 CTL	ERS16327843	this study	10607853	1641339	15%	8 777 369	83%
sRNAseq	sRNA_ICL-RNAi_IP-PTIWI09_T0_JkN_S151	IPptiwi T0 CTL	ERS16327844	this study	11943676	7355448	62%	10 980 105	92%
sRNAseq	sRNA_ICL-RNAi_IP-PTIWI09_T25_JkN_S152	IPptiwi T25 CTL	ERS16327845	this study	7947370	3023379	38%	7 265 026	91%
sRNAseq	sRNA_GTSF1L-RNAi_Input_T0_JkN_S153	Input T0 GTSF1L	ERS16327846	this study	9063379	6263529	69%	8 226 346	91%
sRNAseq	sRNA_GTSF1L-RNAi_Input_T25_JkN_S154	Input T25 GTSF1L	ERS16327847	this study	8426310	3470089	41%	6 949 269	82%
sRNAseq	sRNA_GTSF1L-RNAi_IP-PTIWI09_T0_JkN_S155	IPptiwi T0 GTSF1L	ERS16327848	this study	10815354	7522230	70%	9 961 101	92%
sRNAseq	sRNA_GTSF1L-RNAi_IP-PTIWI09_T25_JkN_S156	IPptiwi T25 GTSF1L	ERS16327849	this study	7531389	5276938	70%	6 930 118	92%
sRNAseq	sRNA_Input_T0_JkN_2023_08_08	sRNA Input T0 rep 1	ERS18352460	this study	17767843	11187298	63%	16 289 704	92%
sRNAseq	sRNA_IP-Gtsf1_T0_JkN_2023_08_08	sRNA IP-Gtsf1 T0 rep 1	ERS18352461	this study	13464334	10152911	75%	12 102 323	90%
sRNAseq	sRNA_Input_2_FLAG_IP-GTSF1_JkN_20240130T082352Z_S158	sRNA Input T0 rep 2	ERS18352462	this study	11729887	7321827	62%	10 596 094	90%
sRNAseq	sRNA_Ouput_2_FLAG_IP-GTSF1_JkN_20240130T082352Z_S159_R1	sRNA IP-FLAG-Gtsf1 T0 rep 2	ERS18352463	this study	9278002	7309134	79%	8 391 858	90%

Supplementary Table S4. Sequencing data and mapping statistics

DNA-seq, RNA-seq and sRNA-seq data were used in this study. For each sequencing sample, the ENA accession is specified, followed by the number of reads sequenced and the mapped reads on the MAC and the MIC reference genomes.
